# Near-Infrared-Triggered “Bridge-and-Attack” Strategy via a Bioinspired Copper–Polyphenol Nanoarchitectonics Platform for Vascular Normalization-Enhanced Cuproptosis-Immunotherapy of Triple-Negative Breast Cancer

**DOI:** 10.34133/bmr.0398

**Published:** 2026-08-03

**Authors:** Xinru Shen, Ying Zhang, Mengyan Shan, Yiqing Kou, Xintao Jia, Mengru Yang, Changxiang Yu, Jingbai Li, Pan Guo, Jiawei Li, Zhidong Liu

**Affiliations:** ^1^ School of Chinese Materia Medica, Tianjin University of Traditional Chinese Medicine, Tianjin 301617, China.; ^2^ State Key Laboratory of Chinese Medicine Modernization, Tianjin 301617, China.; ^3^ Engineering Research Center of Modern Chinese Medicine Discovery and Preparation Technique, Ministry of Education, Tianjin 301617, China.; ^4^ Haihe Laboratory of Modern Chinese Medicine, Tianjin 301617, China.; ^5^ China-Ghana Belt and Road Joint Laboratory on Traditional Medicine, Tianjin University of Traditional Chinese Medicine, Tianjin 301617, China.

## Abstract

Triple-negative breast cancer (TNBC) presents formidable treatment barriers due to dysfunctional vasculature and an immunosuppressive microenvironment. To address these challenges, we engineered a bioinspired near-infrared (NIR)-responsive copper–polyphenol nanoplatform, SCP, to implement a NIR-triggered “bridge-and-attack” therapeutic strategy. This nanoassembly was constructed through the coordination of salvianolic acid B (SAB) with copper ions and further stabilized by a polydopamine (PDA) shell. Upon NIR irradiation, SAB and Cu^2+^ were co-released from SCP, enabling simultaneous vascular remodeling and tumor cell killing. The released SAB promoted vascular normalization, increasing pericyte coverage to 51.6% and alleviating tumor hypoxia, thereby facilitating intratumoral penetration and immune-cell infiltration. Meanwhile, released Cu^2+^, together with PDA-mediated photothermal activation, induced cuproptosis and immunogenic cell death (ICD). This combined remodeling of the tumor microenvironment enhanced CD8^+^ T cell infiltration and achieved a tumor inhibition rate of 88.5% in 4T1 tumor-bearing mice with favorable systemic biosafety. Overall, this interfacial nanomaterial design integrates vascular normalization, photothermal-enhanced cuproptosis, and immunotherapy, providing a promising materials-based strategy for TNBC treatment.

## Introduction

Triple-negative breast cancer (TNBC) is a highly aggressive breast cancer subtype characterized by early metastasis, limited targeted therapies, and disproportionately high mortality [1]. Its therapeutic resistance is closely associated with a highly heterogeneous tumor microenvironment (TME), in which abnormal vasculature, dense extracellular matrix, hypoxia, and immunosuppressive cellular networks jointly restrict drug penetration and antitumor immune activation [2,3]. Therefore, developing TME-modulating therapeutic platforms that can overcome these physical and biological barriers while reducing systemic toxicity is urgently needed to improve TNBC treatment outcomes.

Photothermal therapy (PTT) has emerged as a promising tumor treatment by converting near-infrared (NIR) light into localized hyperthermia through photothermal agents [4]. Recent chemistry and materials studies have further broadened the design of photothermal agents, including NIR-responsive nanoplatforms for synergistic PTT/chemotherapy, excited-state regulation of aromatic sulfone photothermal molecules, and structural optimization of single-component organic phototherapeutic agents to enhance photodynamic therapy (PDT)/PTT performance [5–7]. These advances highlight the importance of rational materials design for improving NIR absorption and photothermal conversion, providing a useful basis for the polydopamine (PDA)-mediated photothermal module used in the present salvianolic acid B (SAB)–Cu@PDA (SCP) system.

In addition to direct tumor ablation, PTT can induce immunogenic cell death (ICD), leading to the release of damage-associated molecular patterns (DAMPs) and activation of antitumor immunity [ 8]. Nevertheless, its efficacy remains constrained by the abnormal vasculature of TNBC, including immature endothelial cells, insufficient pericyte coverage, elevated interstitial fluid pressure, and disorganized blood flow [9]. These abnormalities generate poorly perfused “delivery dead zones,” limit photothermal agent penetration, aggravate hypoxia, and restrict immune-cell infiltration, thereby weakening both PTT-mediated ablation and ICD-driven immune activation [ 10,11].

Vascular normalization strategies address these pathological barriers by restoring vascular integrity and function, thereby improving the TME for therapeutic intervention [12]. Through selective pruning of immature vessels and reinforcement of endothelial junctions, vascular normalization enhances therapeutic penetration and, importantly, facilitates immune-cell infiltration [13,14]. Moreover, by alleviating tumor hypoxia and reducing interstitial fluid pressure, vascular normalization mitigates hypoxia-associated pathological processes and contributes to the reprogramming of the immunosuppressive TME [15]. However, vascular normalization alone has limited antitumor efficacy because it does not directly eradicate malignant cells or sufficiently induce durable antitumor immunity [16,17]. Thus, combining vascular normalization with cytotoxic and immunostimulatory therapies represents a promising strategy for TNBC treatment [18–20]. In this framework, vascular normalization acts as a therapeutic “bridge” that opens access for subsequent tumor-directed “attack” interventions.

In this context, SAB, a natural polyphenolic compound derived from *Salvia miltiorrhiza* [21], has attracted increasing attention as a vascular-regulatory molecule for TME remodeling [22,23]. Recent studies have demonstrated that SAB can restore endothelial junction integrity by modulating Ezh2 expression, thereby promoting tumor vascular normalization [24]. In addition, as a polyphenol, SAB is capable of chelating metal ions to form metal–polyphenol coordination complexes with pH-responsive properties, facilitating the release of therapeutic cargos within the acidic TME [25,26]. Meanwhile, copper ions (Cu^2+^) have emerged as potent anticancer effectors owing to their ability to trigger cuproptosis, a copper-dependent form of regulated cell death [27]. Mechanistically, excessive intracellular copper binds to lipoylated mitochondrial proteins, promotes their aggregation, and induces the loss of iron–sulfur cluster proteins, ultimately leading to proteotoxic stress and mitochondrial dysfunction [28]. Beyond cuproptosis induction, Cu^2+^ can also catalyze Fenton-like reactive oxygen species (ROS) generation under acidic tumor conditions, thereby amplifying oxidative stress-mediated cytotoxicity [29]. However, nonspecific Cu^2+^ leakage and systemic toxicity remain major concerns [30]. Therefore, SAB serves as a dual-functional building block: a vascular-normalizing pharmacophore to establish the therapeutic “bridge” and a polyphenolic coordination scaffold to stabilize Cu^2+^ during circulation while enabling pH-responsive release for subsequent cuproptosis/oxidative “attack”.

**Fig. 1.**
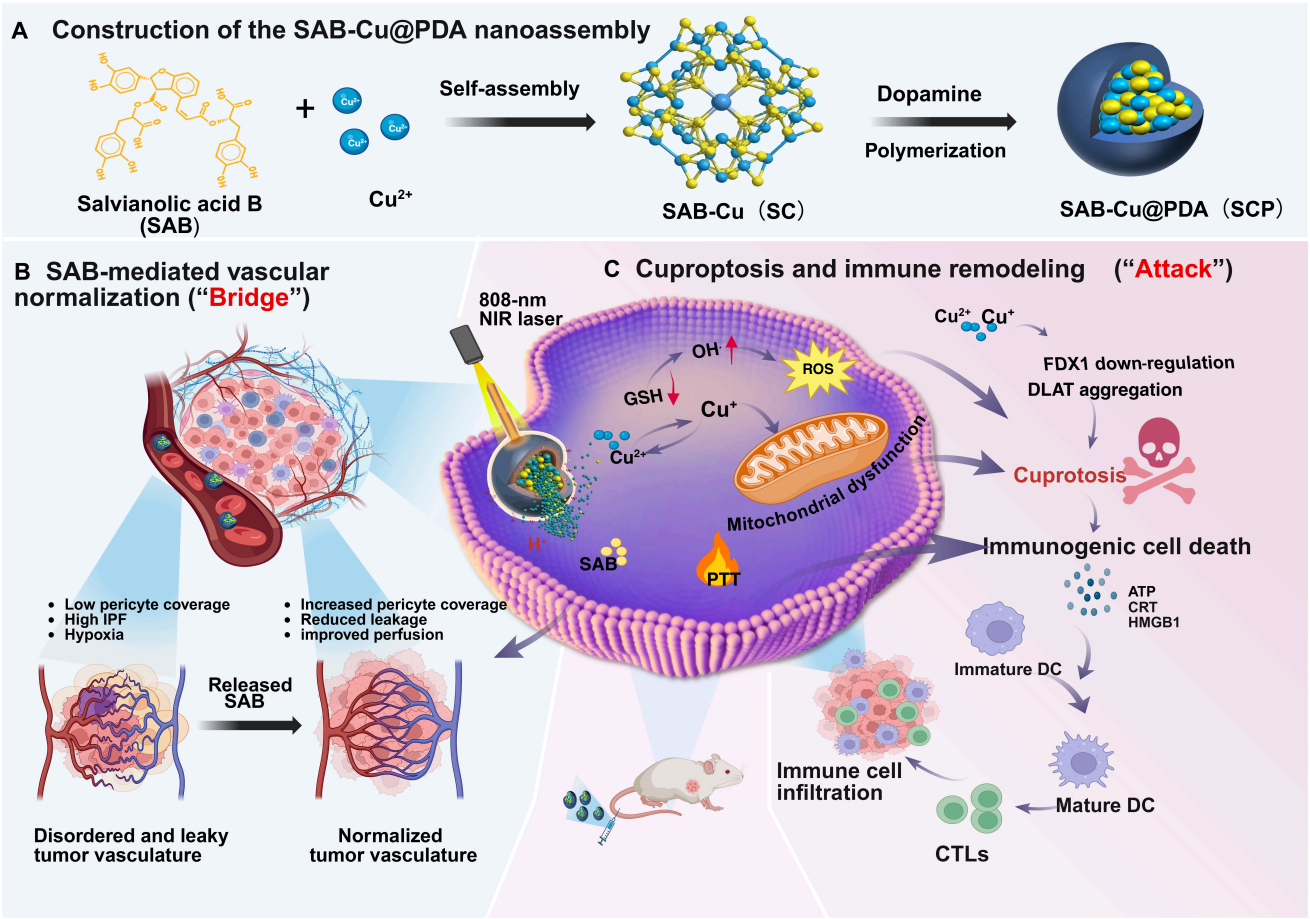
Schematic illustration of the construction and NIR-triggered “bridge-and-attack” therapeutic mechanism of the SCP. (A) Construction of the SAB-Cu@PDA nanoassembly via SAB and Cu^2+^ self-assembly followed by dopamine polymerization. (B) SAB-mediated vascular normalization (“bridge”) promoting increased pericyte coverage, reduced leakage, improved perfusion, and hypoxia alleviation. (C) Cuproptosis and immune remodeling (“attack”) induced by NIR irradiation, leading to ROS generation, GSH depletion, mitochondrial dysfunction, ICD, and immune cell infiltration.

In this study, we developed a bioinspired NIR-responsive nanoassembly, SAB–Cu@PDA (SCP), based on the coordination between the plant-derived natural polyphenol SAB and Cu^2+^, together with a mussel-inspired PDA coating. This bioinspired design enables the formation of a stable coordination nanoassembly with NIR absorption and stimuli-responsive release properties, thereby supporting the proposed “bridge-and-attack” therapeutic strategy. The PDA shell acts as a biomimetic gatekeeper, enabling efficient photothermal conversion and NIR-triggered co-release of SAB and Cu^2+^. In this design, released SAB serves as the “bridge” component by promoting vascular normalization and facilitating intratumoral penetration, while released Cu^2+^, together with PDA-mediated photothermal activation, acts as the “attack” component by inducing dihydrolipoamide S-acetyltransferase (DLAT)-associated cuproptosis and ICD. Through these coupled effects, SCP remodels the TNBC microenvironment and enhances antitumor immune activation. The overall design of the SCP nanoassembly and its NIR-triggered “bridge-and-attack” therapeutic mechanism are schematically illustrated in Fig. 1.

## Materials and Methods

### Materials

SAB was obtained from Shanghai Yuan Ye Biotechnology Co. Ltd. Copper nitrate Cu (NO₃)₂ was sourced from Shanghai Yien Chemical Technology Co. Ltd. Dopamine was acquired from Sigma-Aldrich Trading Co. Ltd. o-Phenylenediamine (OPD, 5,5′-dithiobis (2-nitrobenzoic acid) (DTNB), 3,3′,5,5′-tetramethylbenzidine (TMB), and Rhodamine B were bought from Aladdin Scientific Corp. The adenosine triphosphate (ATP) analysis kit, enhanced mitochondrial membrane potential assay kit (JC-1), and Calcein/PI Cell Viability and Cytotoxicity Assay Kit were purchased from Beyotime Biotech Inc. The HMGB1 assay kit was sourced from Shanghai Enzyme-linked Biotechnology Co. Ltd. The Annexin V-FITC/PI Apoptosis Detection Kit was purchased from Beijing Solarbio Science & Technology Co. Ltd. The Cell Counting Kit-8 (CCK-8) assay kit was obtained from Tianjin Youkang Biotechnology Co. Ltd. Aspartate aminotransferase (AST), creatine kinase (CK), and creatinine (CRE) detection kits were purchased from Elabscience Biotechnology Co. Ltd. Antibodies used in the study include CD8a–allophycocyanin (APC), CD45–phycoerythrin (PE), CD11C–APC–eFluor 780, CD80–Super Bright 600, CD3E–fluorescein isothiocyanate (FITC), and Fixable Viability Dye eFluor 506, all purchased from Thermo Fisher Scientific. CD86–eFluor 450 was purchased from BioLegend. DLAT and FDX1 antibodies, CoraLite488-conjugated Goat Anti-Rabbit IgG (H+L), and CoraLite594-conjugated Goat Anti-Rabbit IgG (H+L) were obtained from Proteintech. RPMI 1640, Dulbecco’s modified Eagle’s medium (DMEM), fetal bovine serum (FBS), streptomycin/penicillin, and other cell culture reagents were acquired from Gibco.

### Cell lines

4T1, 3T3, and human umbilical vein endothelial cells (HUVECs) (Wuhan Procell) were cultured in RPMI 1640 (4T1) or DMEM (3T3, HUVECs) supplemented with 10% FBS and 1% antibiotic–antimycotic at 37 °C in 5% CO₂.

### Animals

Healthy female BALB/c mice were purchased from SPF (Beijing) Biotechnology Co. Ltd.

### Synthesis of SCP nanoparticles

SC (SAB–Cu core) was synthesized by stirring SAB (7.2 mg) and copper nitrate (9.5 mg) in an alkaline aqueous solution (room temperature, 24 h). The product was collected via centrifugation (12,000 rpm, 20 min), washed, and re-dispersed in tris–HCl buffer. Subsequently, dopamine hydrochloride (3.5 mg) was added and stirred in the dark for 3 h. Finally, SCP was obtained after centrifugation, washing, and ultrasonic dispersion.

### Characterization

Particle size and zeta potential of SCP were measured using a Zetasizer (Malvern Instruments, UK). SCP was characterized by transmission electron microscopy (TEM) (JEM-1200EX, JEOL, Japan). X-ray diffraction (XRD) (Bruker D8 ADVANCE, Germany) was performed at 40 kV and 40 mA using Cu Kα (λ = 1.5406 Å) and Co Kα (λ = 1.79026 Å) radiation. Energy-dispersive x-ray spectroscopy (EDS) was conducted via a FEI Talos F200X. Copper ion analysis and elemental composition were determined by inductively coupled plasma mass spectrometry (ICP-MS) (Agilent 7800, USA) and XPS (Thermo ESCALAB 250Xi, USA), respectively. Ultraviolet–visible (UV–vis) (MAPADA UV-6100, China) and Fourier transform infrared (FT-IR) (Bruker, Germany) spectra were recorded to evaluate optical and structural properties.

### Drug release assay in vitro

The pH- and photothermal-responsive release of SCP was evaluated via dialysis. SCP dispersion (1 ml, 50 μg/ml) was sealed in dialysis bags and immersed in 50 ml of phosphate-buffered saline (PBS) (pH 7.4 or 6.5) at 37 °C under 100 rpm stirring. For photothermal evaluation, the NIR group received 5 min of laser irradiation before each sampling, with a non-irradiated group as the control. At scheduled intervals (10 min to 24 h), 1 ml of the medium was collected and replaced with fresh prewarmed PBS. Released Cu^2+^ was quantified via the dithizone colorimetric assay, while the released SAB concentration was quantified by UV–vis according to a pre-established SAB standard curve, and cumulative release was calculated using the following formula:

$$\text{Cumulative release}=\frac{C_{t}V+\sum_{i=1}^{i-1}C_{i}v}{M}\times 100\%$$ (1)

where *C_t_* is the Cu^2+^ or SAB concentration at time *t* (μg/ml), *V* is the total release medium volume (ml), *v* is the sampling volume (ml), and *M* is the mass of drug in the dialysis bag. All experiments were performed in triplicate (*n* = 3).

### Photothermal conversion efficiency measurement

The photothermal conversion efficiency (η) of SCP (400 μg/ml, 1 ml) was determined under 808-nm laser irradiation (2 W/cm^2^, 5 min). Temperature changes were monitored at regular intervals via an infrared thermal imager. The η value was calculated using the following formula (*n* = 3)

$$\eta =\frac{\mathrm{hS}\left(T_{\max}-T_{\mathrm{sur}}\right)-Q_{\mathrm{dis}}}{I\left(1-10^{-A_{808}}\right)}$$ (2)

$$\mathrm{hS}=\frac{m_{A}C_{\text{water}}}{\tau_{s}}$$ (3)

$$Q_{\mathrm{dis}}=hS\left(T_{\max,\text{Water}}-T_{\mathrm{sur},\text{Water}}\right)$$ (4)

$$\theta =\frac{T-T_{\mathrm{sur}}}{T_{\max}-T_{\mathrm{sur}}}$$ (5)

$$t=-\tau_{s}\ln\left(\theta \right)$$ (6)

where *h* and *S* are the heat transfer coefficient and container surface area, respectively; *T_max_* and *T*_sur_ are the equilibrium and ambient temperatures, respectively; *Q*_dis_ is the heat dissipation from the solvent and container; *I* is the incident laser power; *A*_808_ is the sample absorbance at 808 nm; *m*_A_ and *C*_water_ represent the solution mass and the specific heat capacity of water; and $\tau_{s}$ is the system time constant.

### Cellular uptake of SCP nanoparticles

To evaluate the cellular uptake and photothermal response of SCP, 4T1 cells were incubated with Rho B-labeled SCP (since SCP is non-fluorescent) for 2 or 4 h. The NIR group was irradiated (808 nm, 2.0 W/cm^2^, 5 min) during incubation. Nuclei were stained with Hoechst 33342, and imaging was performed via confocal laser scanning microscopy (CLSM). Quantitative uptake was further analyzed by flow cytometry. The excitation and emission wavelengths were set as follows: Hoechst 33342 (blue): λ_ex_ = 346 nm, λ_em_ = 460 nm; Rho B (red): λ_ex_ = 556 nm, λ_em_ = 570 nm.

### RNA extraction and quantitative real-time PCR

Total RNA was extracted using Trizol reagent (Invitrogen, USA) and quantified via a NanoDrop 2000c spectrophotometer (Thermo Fisher, USA). cDNA was synthesized using FastKing gDNA Dispelling RT SuperMix (TIANGEN BIOTECH, China). Quantitative real-time polymerase chain reaction (qRT-PCR) was performed with SYBR Green Master Mix (TIANGEN BIOTECH) on a BIOER real-time PCR system. Relative gene expression was normalized to glyceraldehyde-3-phosphate dehydrogenase (GAPDH) using the 2^−ΔΔCt^ method. Primer sequences are listed below:

FDX1-F: 5′-ACAGTCCACTTCAAGAACCGAG-3'

FDX1-R: 5′-CATCTAGCAGAGAGTCGCCAAT-3'

LIAS-F: 5′-CCTTACCTGCCTCAAGCAATC-3'

LIAS-R: 5′-TCTCATAGCCCACACAACAGAC-3'

GAPDH-F: 5′-GACACTGAGCAAGAGAGGCCCTA-3'

GAPDH-R: 5′-TGGGATGGAAATTGTGAGGGA-3'

### Cytotoxicity assay

3T3 and 4T1 cells were seeded in 96-well plates (1 × 10^4^ cells per well) and cultured for 24 h. After washing with PBS, cells were incubated with drug-containing media for another 24 h. For the photothermal group, cells were irradiated (5 min) 4 h post-treatment. Cell viability was then assessed via CCK-8 assay after PBS washing. The survival rate was calculated as follows:

$$\text{Cell viability}\left(\%\right)=\left[\frac{A_{s}-A_{b}}{A_{c}-A_{b}}\right]\times 100\%$$ (7)

where *A*_s_ is the absorbance of the treatment group, *A*_c_ is the absorbance of the control group, and *A*_b_ is the Absorbance of the blank group.

### Confocal microscopy was used to further investigate the cytotoxicity of SCP

Cells were seeded on coverslips in 12-well plates (5 × 10^4^ cells per well). Following attachment, cells were assigned to 4 groups: (a) Control, (b) Laser only, (c) SCP (50 μg/ml), and (d) SCP + Laser (808 nm, 2 W/cm^2^, 5 min, 4 h post-treatment). After 24 h, live/dead staining was performed using calcein-AM/propidium iodide (PI) co-incubation (30 min). Fluorescence microscopy was employed to visualize green (calcein-AM, excitation/emission = 494/517 nm) and red (PI, excitation/emission = 535/617 nm) fluorescence. Fluorescence images were acquired using a STELLARIS 8 laser scanning confocal microscope (Leica Microsystems, Wetzlar, Germany).

### Immunofluorescence assay

Cells were seeded on coverslips in 6-well plates and incubated overnight. For immunofluorescence, cells were fixed [4% paraformaldehyde (PFA), 10 min], permeabilized (0.25% Triton X-100, 10 min), and blocked [3% bovine serum albumin (BSA), 1 h]. Primary antibodies against DLAT and FDX1 (1:500) were incubated at 4 °C overnight. After washing thrice with 0.1% phosphate-buffered saline with Tween-20 (PBS-T), cells were treated with fluorescent secondary antibodies (37 °C, 1 h). Coverslips were then mounted with 4′,6-diamidino-2-phenylindole (DAPI)-containing medium and imaged via CLSM.

### In vivo antitumor efficacy

When tumor volumes reached ≈100 mm^3^, 30 tumor-bearing mice were randomized into 6 groups (*n* = 5): Saline, Saline + NIR, SAB, SC, SCP, and SCP + NIR. Treatments were administered via tail vein every 3 d for 6 doses (SAB-equivalent dose: 1 mg/kg). This SAB-equivalent dose was selected because previous SAB vascular normalization studies mainly used free SAB by oral gavage, whereas the present study used an intravenously administered SCP nanoassembly. Moreover, because SAB and copper were co-assembled in the SC core, increasing the SAB-equivalent dose would also increase copper exposure, potentially elevating copper-related systemic toxicity. Therefore, this dose was chosen to balance vascular-regulatory efficacy, intravenous nanoformulation delivery, and copper-related safety. For NIR groups, tumors were irradiated (808 nm, 1.5 W/cm^2^, 5 min) 24 h post-injection. Body weight and tumor volume (V) were recorded every 2 d. On day 19, tumors were harvested for hematoxylin and eosin (H&E) staining, TUNEL (terminal deoxynucleotidyl transferase-mediated deoxyuridine triphosphate nick end labeling) assay, and histological examination.

$$V=\frac{1}{2}\times a\times b^{2}$$ (8)

where *a* is the longest diameter (mm) and *b* is the shortest diameter perpendicular to length (mm).

### In vivo biodistribution of SCP

To evaluate biodistribution, 4T1 tumor-bearing BALB/c mice (≈ 200 mm^3^) were intravenously injected with indocyanine green (ICG) or SCP-ICG (0.5 mg/kg ICG-equivalent). Mice were anesthetized and imaged at 4, 12, and 24 h post-injection using an IVIS Spectrum system (PerkinElmer, USA). At 24 h, mice were euthanized; major organs and tumors were harvested for ex vivo fluorescence imaging (*n* = 3).

### Scanning electron microscopy of tumor vasculature

Tumor samples were washed with PBS, fixed in 3% glutaraldehyde (2 h), and rinsed with ultrapure water (3×, 10 min each). Samples were post-fixed in 1% osmium tetroxide (1 to 2 h), rinsed again, and dehydrated through a graded ethanol series. Following critical point drying, tumors were mounted on conductive stubs and sputter-coated with gold. Scanning electron microscopy (SEM) imaging was performed using a JSM-IT700HR (JEOL Ltd., Japan).

### Flow cytometric analysis of immune cells

Following treatment, spleen and tumor tissues were harvested (*n* = 3). Tumors were digested in Hanks’ balanced salt solution (HBSS) containing collagenase IV (1 mg/ml) and deoxyribonuclease (DNase) I (20 U/ml) at 37 °C (80 rpm, 30 min) and then filtered (70-μm strainer) to obtain single-cell suspensions. Splenocytes were isolated via mechanical grinding and erythrocyte lysis. Cells were blocked (Fc blocker, 4 °C, 5 min) and stained with fluorochrome-labeled antibodies. Flow cytometry (BD FACSCelesta) was used to quantify tumor-infiltrating dendritic cells (DCs) (CD45^+^CD11c^+^CD80^+^CD86^+^) and cytotoxic T lymphocytes (CTLs) (CD45^+^CD3^+^CD8^+^).

### Immunofluorescence staining of tumor tissue

Tumor tissues were fixed in 4% PFA (24 h), dehydrated (30% sucrose), and embedded in optimal cutting temperature compound (OCT) for cryosectioning (5 to 10 μm). Following heat-induced antigen retrieval (10 mM sodium citrate, pH 6.0), sections were permeabilized (0.5% Triton X-100, 15 min) and blocked (5% goat serum/1% BSA, 1 h). Primary antibodies against DLAT, FDX1 (1:200), hypoxia-inducible factor-1α (HIF-1α), CRT, and HMGB1 (1:100) were incubated at 4 °C overnight, followed by species-matched Alexa Fluor secondary antibodies (1:500, 1 h) and DAPI counterstaining (10 min). Slides were mounted and imaged via confocal microscopy. Quantitative analysis was performed on 3 random fields per section using ImageJ.

### Vascular maturation analysis

Double immunofluorescence staining was performed using species-specific primary antibodies: mouse anti-CD31 (1:200) + rabbit anti-α-SMA (smooth muscle actin) (1:100) or rabbit anti-NG2 (1:200) incubated overnight at 4 °C, followed by Alexa Fluor-conjugated secondary antibodies (goat anti-mouse immunoglobulin G (IgG)-546, red; goat anti-rabbit IgG-488, green; 1:500, 1 h in dark). After DAPI counterstaining, sequential confocal imaging (CD31: excitation/emission 556/573 nm; α-SMA/NG2: 495/519 nm) quantified smooth muscle cell coverage (α-SMA^+^) or pericyte coverage (NG2+) as:

$$\text{Coverage}\;\left(\%\right)=\frac{\text{Colocalized area}}{\text{Total}\;\mathrm{CD}31^{+}\;\text{area}}$$ (9)

### Fluorescence intensity

Fluorescence intensity was quantified using ImageJ software. For each group, multiple randomly selected fields were analyzed under identical imaging and thresholding conditions, and the results were expressed as relative fluorescence intensity or positive staining area, as indicated in the corresponding figure legends.

## Results

### Synthesis and structural characterization of SCP

**Fig. 2.**
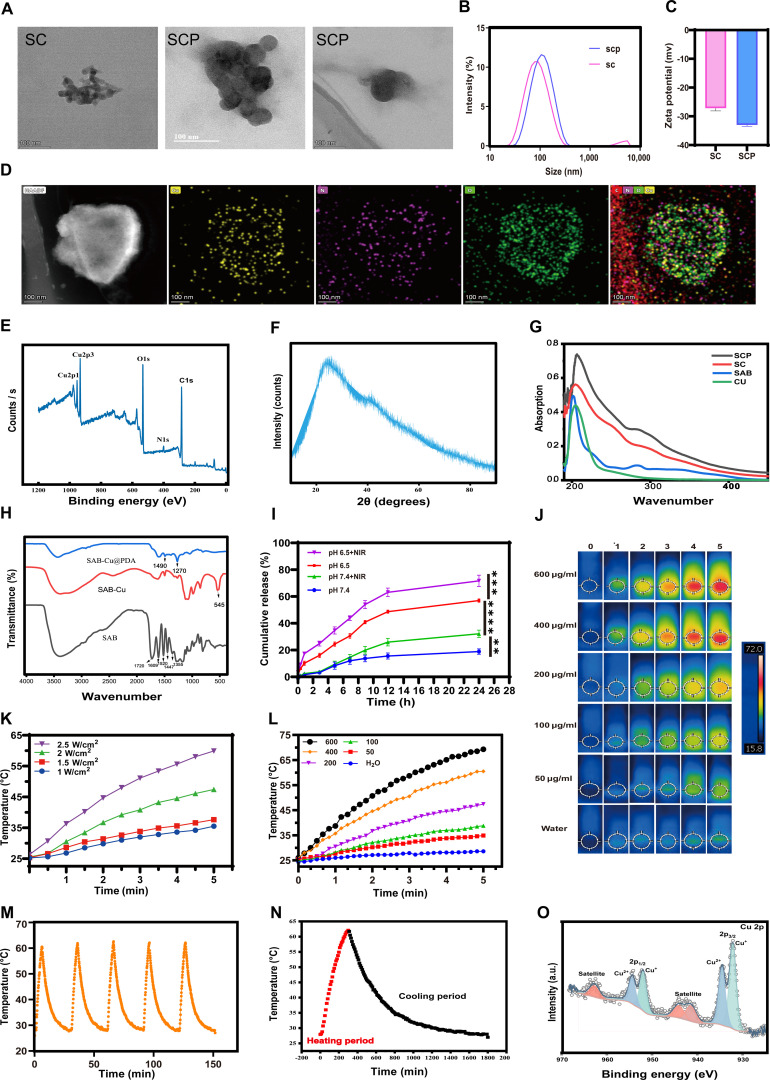
Synthesis, physicochemical characterization, responsive release, and photothermal properties of the SCP nanoassembly. (A) Transmission electron microscopy (TEM) images of SC and SCP. (Scale bar, 100 nm.) (B) Particle size distributions of SC and SCP. (C) Zeta potentials of SC and SCP. (D) Elemental mapping images of SCP. (Scale bar, 100 nm.) (E) X-ray photoelectron spectroscopy (XPS) survey spectrum of SCP. (F) X-ray diffraction (XRD) pattern of SCP. (G) UV–vis absorption spectra of Cu^2+^, SAB, SC, and SCP. (H) FT-IR spectra of SAB, SC, and SCP. (I) Cumulative Cu^2+^ release profiles from SCP under different pH conditions with or without 808-nm NIR irradiation (*n* = 3). (J) Infrared thermal images of SCP at different concentrations under 808-nm NIR irradiation. (K) Photothermal heating curves of SCP under different laser power densities. (L) Photothermal heating curves of SCP at different concentrations. (M) Temperature changes of SCP during 5 laser on/off irradiation cycles. (N) Heating and cooling curve of SCP for photothermal conversion efficiency calculation. (O) High-resolution XPS analysis of Cu valence states in SCP after acid treatment. Data are presented as mean ± SD (*n* = 3). Statistical comparisons among groups at 24 h were analyzed by 2-way analysis of variance (ANOVA) followed by Tukey’s multiple-comparisons test. ns, not significant; *P < 0.05, **P < 0.01, ***P < 0.001, ****P < 0.0001.

The multifunctional nanoplatform SCP, denoted as SCP, was prepared through a bioinspired self-assembly strategy. SAB was first coordinated with Cu^2+^ in aqueous solution to form the SC, followed by oxidative polymerization of dopamine to generate the PDA shell (Fig. 2A). During the initial formulation optimization, different SAB^2+^ feeding mass ratios were compared to optimize the SC coordination core. Among the tested ratios, 1:5 produced uniform nanoparticles with a suitable particle size, low polydispersity index (PDI), and balanced encapsulation efficiencies of both SAB and Cu, whereas 1:1 resulted in severe aggregation and 1:9 showed increased particle size and PDI. Therefore, the SAB^2+^ feeding mass ratio of 1:5 was selected for subsequent PDA coating and biological studies (Fig. S1). The PDA coating level was further optimized by varying the dopamine feeding amount during the coating process. Among the tested SAB feeding mass ratios, 1:0.5 showed the best overall performance, with relatively low PDI, efficient NIR-induced temperature elevation, and favorable Cu^2+^ release behavior. Excessive dopamine may cause self-polymerization in solution and reduce coating uniformity, whereas insufficient dopamine may weaken the photothermal effect. Therefore, the SAB feeding mass ratio of 1:0.5 was selected for subsequent SCP preparation (Fig. S2).

After formulation optimization, DLS showed an increased hydrodynamic diameter after PDA coating, preliminarily confirming the formation of SCP (Fig. 2B). Consistently, the zeta potential shifted from −27.17 ± 0.78 mV for SC to −33.03 ± 0.46 mV for SCP, which was attributed to the abundant deprotonated phenolic/hydroxyl groups in the PDA layer at physiological pH (Fig. 2C) [31]. Elemental mapping confirmed the homogeneous distribution of Cu, C, N, and O within SCP (Fig. 2D). In addition, the x-ray photoelectron spectroscopy (XPS) survey spectrum verified the elemental composition of SCP, further supporting the successful construction of the SCP nanoassembly (Fig. 2E). The XRD pattern of SCP showed no obvious crystalline peaks but a broad amorphous halo, indicating the amorphous nature of the SC coordination framework (Fig. 2F). Such an amorphous structure may favor pH-responsive dissociation in the acidic TME.

​ The stepwise structural evolution was further investigated by UV–vis spectroscopy (Fig. 2G). Free SAB displayed characteristic absorption bands at 202 and 283 nm, while coordination with Cu^2+^ induced redshifts to 205 nm and a shoulder around 300 nm, respectively, suggesting the formation of the SC coordination core [32]. After PDA coating, the absorption band further shifted to 207 nm, confirming the successful construction of SCP. In addition, SCP exhibited concentration-dependent NIR absorption, supporting its potential for photothermal activation (Fig. S3).

FT-IR spectroscopy further verified the coordination interaction and PDA coating (Fig. 2H). Compared with free SAB, SC exhibited a new band at 545 cm^−1^, corresponding to Cu–O stretching, indicating successful coordination between Cu^2+^ and SAB [33]. After PDA coating, characteristic bands at 1,270 and 1,490 cm^−1^ appeared in SCP, attributable to aromatic/C–N related vibrations and N–H bending of PDA, respectively, confirming the formation of the PDA shell. Together, these results demonstrate the successful construction of the SCP nanoplatform. SCP also showed good colloidal stability, with negligible changes in hydrodynamic size and morphology after incubation in DI water, PBS, and 10% FBS for 7 d (Fig. S4). In addition, the hemolysis assay confirmed its favorable hemocompatibility (Fig. S5), supporting its potential for systemic administration.

### Responsive cargo release and superior photothermal efficiency

The pH- and NIR-responsive co-release behaviors of Cu^2+^ and SAB from SCP were evaluated to verify its on-demand delivery capability. Under physiological pH 7.4, both Cu^2+^ and SAB showed limited release, indicating good formulation stability. In contrast, acidic conditions markedly accelerated the release of both components, and 808-nm NIR irradiation further enhanced their release. These results demonstrate that SCP enables pH/NIR-responsive co-release of SAB and Cu^2+^ under tumor microenvironment-mimicking conditions (Fig. 2I and Fig. S6).

This dual-triggered release behavior can be attributed to acid-induced weakening of the SAB–Cu^2+^ coordination and PDA shell, together with NIR-mediated photothermal activation that facilitates matrix loosening and ion diffusion [34]. Such a controlled release profile is favorable for reducing premature Cu^2+^ leakage during circulation while promoting tumor-localized Cu^2+^ accumulation for cuproptosis-based therapy. Owing to its broad NIR absorption, the photothermal performance of SCP was evaluated under 808-nm laser irradiation. SCP dispersions showed a rapid temperature increase, whereas pure water exhibited negligible heating (Fig. 2J). The photothermal effect was both concentration- and power density-dependent, indicating controllable thermal output (Fig. 2K and L). SCP also maintained stable heating performance over 5 laser on/off cycles, demonstrating good photothermal stability (Fig. 2M). Based on the heating–cooling profile, the heat transfer time constant ($\tau_{s}$) was calculated as 405.8 s, and the photothermal conversion efficiency (η) was determined to be 59.5% (Fig. 2N and Fig. S7). These results confirm the efficient and stable photothermal capability of SCP, supporting its use for NIR-triggered release and photothermal-enhanced cuproptosis therapy.

To further verify the contribution of the PDA shell to the photothermal performance, SC, the PDA-free SAB–Cu coordination core, was used as a control. Under 808-nm laser irradiation at 2 W/cm^2^ and at the same concentration of 400 μg/ml, SCP showed a rapid and pronounced temperature increase, whereas SC exhibited only a slight temperature elevation comparable to that of H₂O (Fig. S8). These results demonstrate that the SAB–Cu coordination core contributes minimally to photothermal heating, and that the efficient NIR-responsive photothermal performance of SCP is mainly derived from the PDA shell.

### Chemodynamic activation and redox microenvironment regulation

The copper valence state in SCP was analyzed by high-resolution x-ray photoelectron spectroscopy (XPS). In the intact formulation, the Cu 2p peaks at 933.54 and 953.31 eV were assigned to Cu^+^ 2p₃/₂ and Cu^+^ 2p₁/₂, respectively, indicating the reduction of coordinated Cu^2+^ to Cu^+^ during SAB–Cu assembly (Fig. S9). This conversion is attributed to the strong electron affinity of copper and electron transfer from the highly conjugated SAB framework [32]. After incubation under acidic TME-mimicking conditions, SCP exhibited a mixed Cu^+^/Cu^2+^ state, with Cu^+^ accounting for 63.28% and Cu^2+^ for 36.27%, accompanied by characteristic Cu^2+^ satellite peaks (Fig. 2O). This valence conversion suggests acid-triggered coordination weakening and Cu^+^ oxidation, providing a redox-active Cu^+^/Cu^2+^ cycle for chemodynamic activation.

The Fenton-like catalytic activity of SCP was then evaluated using H₂O₂ and OPD as indicators. SCP effectively catalyzed H₂O₂ decomposition to generate ·OH, as evidenced by the characteristic oxidized OPD absorption at 417 nm (Fig. S10). Notably, 808-nm NIR irradiation further enhanced ·OH production, likely due to photothermal acceleration of Cu-mediated Fenton-like reactions. The ·OH generation increased with SCP concentration and irradiation time, confirming a controllable and stimuli-responsive ROS-generating capability.

In the TME, overexpressed glutathione (GSH) serves as a major antioxidant barrier that scavenges ROS and weakens chemodynamic efficacy. The GSH depletion ability of SCP was evaluated using the DTNB assay (Fig. S11). At pH 6.5, SCP induced concentration-dependent GSH consumption, with near-complete depletion at 400 μg/ml. Notably, SCP showed stronger GSH depletion than the uncoated SC core under acidic conditions, which may be attributed to Cu^2+^/Cu^+^ redox cycling and thiol-mediated interactions with the PDA shell [35]. In contrast, minimal GSH consumption was observed at pH 7.4, indicating good stability of SCP under physiological conditions. These results demonstrate the pH-activated ability of SCP to weaken antioxidant defenses and amplify ROS-mediated oxidative stress.

### Cellular internalization and NIR-enhanced tumor cell ablation

**Fig. 3.**
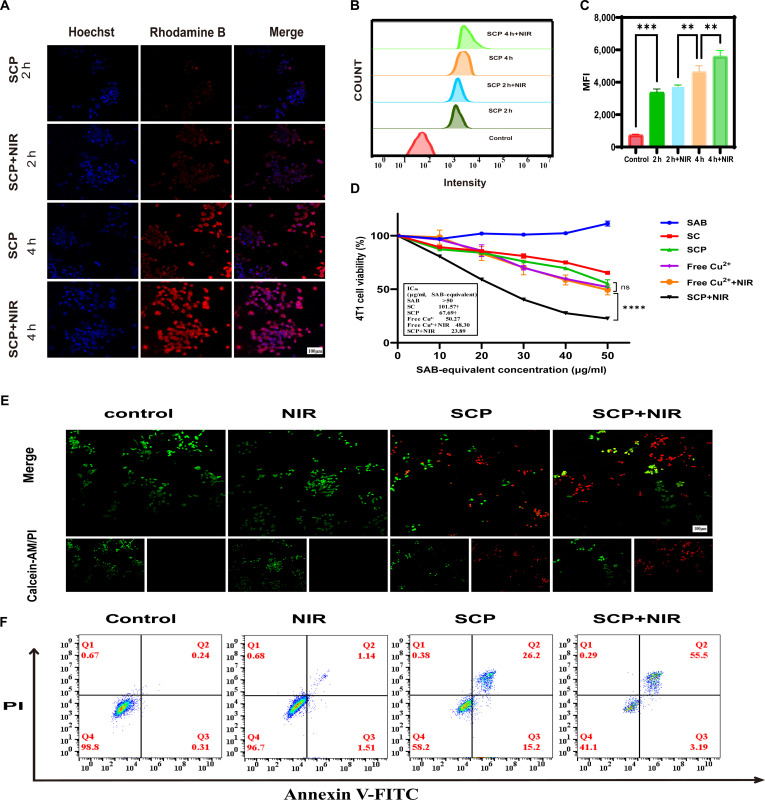
Cellular internalization and synergistic cytotoxicity of SCP in vitro. (A) CLSM images of 4T1 cells incubated with Rhodamine B-labeled SCP for 2 and 4 h with or without NIR irradiation. (Scale bar, 100 μm.) (B) Flow cytometric analysis of SCP uptake in 4T1 cells and (C) corresponding mean fluorescence intensity (MFI) quantification (*n* = 3). (D) Cell viability of 4T1 cells after treatment with SAB, SC, SCP, free Cu^2+^, free Cu^2+^ + NIR, and SCP + NIR at different SAB-equivalent concentrations (*n* = 3). IC_50_ values were calculated by nonlinear dose–response regression and expressed as SAB-equivalent concentration. Values exceeding the highest tested concentration were estimated by model extrapolation. (E) Live/dead staining images of 4T1 cells after different treatments. (Scale bar, 100 μm.) (F) Representative Annexin V-FITC/PI flow cytometry plots of 4T1 cells after different treatments. Data are presented as mean ± SD. Statistical significance in (C) was analyzed by one-way ANOVA followed by Tukey’s multiple-comparisons test. Statistical significance in (D) was analyzed by 2-way ANOVA followed by Tukey’s multiple-comparisons test. ns, not significant; **P < 0.01, ***P < 0.001, ****P < 0.0001.

To evaluate intracellular delivery, Rhodamine B-labeled SCP was incubated with 4T1 cells. CLSM images showed time-dependent intracellular fluorescence, with stronger signals at 4 h than at 2 h, indicating efficient cellular uptake (Fig. 3A and Fig. S12). NIR irradiation further enhanced fluorescence intensity, likely due to PDA-mediated local photothermal effects that transiently increase membrane permeability [36]. Flow cytometry confirmed the time-dependent and NIR-enhanced internalization of SCP (Fig. 3B and C).

The cytotoxicity of SCP was then assessed by CCK-8 assay. SCP showed negligible toxicity toward normal cells, with cell viability remaining above 90% at 0 to 50 μg/ml (Fig. S13). In contrast, free Cu^2+^, SC, and SCP induced concentration-dependent cytotoxicity in 4T1 cells, whereas free SAB showed limited inhibitory effects (Fig. 3D). Free Cu^2+^ exhibited higher cytotoxicity than SCP under non-irradiated conditions. In addition, no significant difference was observed between free Cu^2+^ and free Cu^2+^ + NIR groups. By comparison, SCP combined with NIR irradiation resulted in the most pronounced cytotoxicity and the lowest IC₅₀ value among all treatment groups.

Calcein-AM/PI staining further verified the enhanced tumoricidal activity of SCP + NIR. Control and NIR-only groups showed predominantly green fluorescence, whereas SCP + NIR treatment caused extensive red PI staining, indicating severe cell damage (Fig. 3E and Fig. S14). Consistently, Annexin V-FITC/PI flow cytometry revealed a substantial increase in late apoptotic/necrotic cells after SCP + NIR treatment (Fig. 3F). These results demonstrate that NIR-activated SCP efficiently enters 4T1 cells and induces potent chemo-photothermal cytotoxicity.

### In vitro modulation of endothelial function and angiogenic morphogenesis

**Fig. 4.**
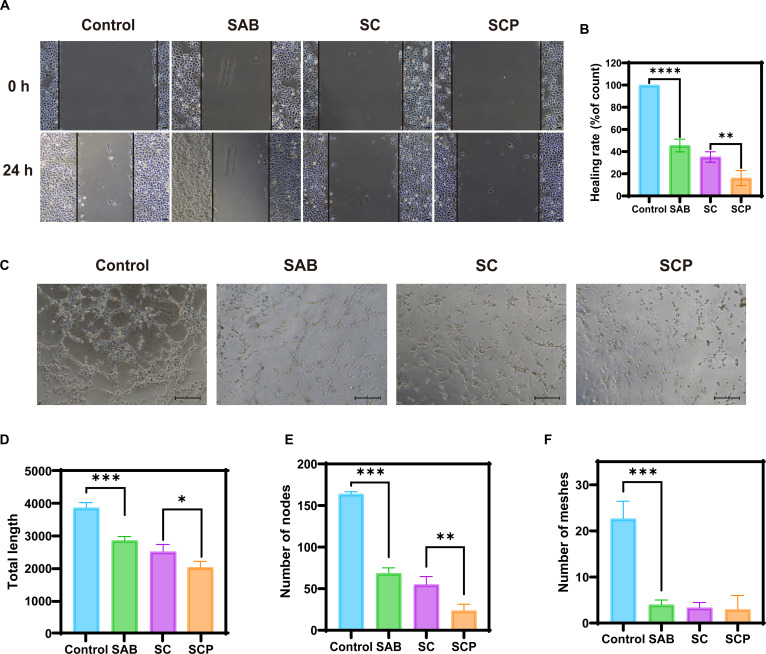
In vitro validation of the vascular “bridge” effect of SCP. (A) Representative images of the tumor-conditioned HUVEC scratch wound assay after different treatments. Conditioned medium collected from treated 4T1 cells was used to stimulate HUVEC migration. (Scale bar, 50 μm.) (B) Quantification of the wound closure rate (*n* = 3). (C) Representative HUVEC tube formation images after different treatments. (Scale bar, 100 μm.) (D to F) Quantification of total tube length, number of nodes, and number of meshes, respectively (*n* = 3). Data are presented as mean ± SD. Statistical significance was analyzed by one-way ANOVA followed by Tukey’s multiple-comparisons test. ns, not significant; *P < 0.05, **P < 0.01, ***P < 0.001, ****P < 0.0001.

To evaluate the vascular “bridge” potential of SCP under tumor microenvironment-mimicking conditions, a tumor-conditioned HUVEC scratch wound assay was performed. Conditioned medium collected from 4T1 cells after different treatments was used to stimulate HUVEC migration, because tumor cell-derived soluble factors are important regulators of endothelial migration in the TME. Compared with the control conditioned medium, conditioned medium from SAB-, SC-, and SCP-treated 4T1 cells reduced HUVEC wound closure, with SCP showing the strongest inhibitory effect (Fig. 4A and B). These results suggest that SCP suppresses endothelial migration under tumor-associated paracrine stimulation, supporting its potential role in vascular regulation and tumor vascular normalization. The effect of SCP on angiogenic morphogenesis was further assessed using a tube formation assay. Compared with the well-organized capillary-like networks in the control group, SAB-containing formulations disrupted tubular structure formation. Among them, SCP caused the most pronounced impairment, as reflected by reduced total tube length, branch points, and mesh area (Fig. 4C to F).

These results indicate that SCP can modulate key endothelial behaviors involved in pathological angiogenesis, including migration and tubular assembly. This in vitro vascular-regulatory effect supports its potential to remodel abnormal tumor vasculature and facilitate subsequent therapeutic penetration.

### Mechanistic elucidation of NIR-triggered cuproptosis and oxidative damage

**Fig. 5.**
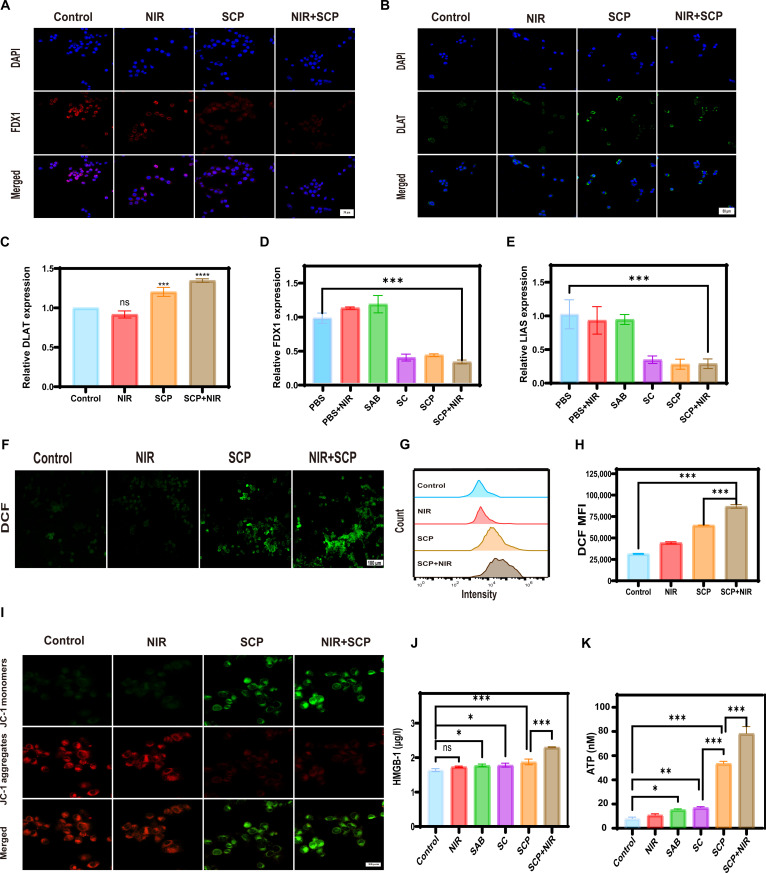
In vitro validation of SCP-induced cuproptosis-associated oxidative damage and ICD. (A) FDX1 and (B) DLAT immunofluorescence images of 4T1 cells after different treatments. (Scale bar, 50 μm.) (C) Quantification of DLAT aggregation based on confocal images (*n* = 3). (D and E) qPCR analysis of FDX1 and LIAS expression (*n* = 3). (F) DCFH-DA fluorescence images and (G) flow cytometric histograms showing intracellular ROS generation. (Scale bar, 100 μm.) (H) Quantitative analysis of ROS levels based on 2ʹ,7ʹ-dichlorofluorescein (DCF) MFI (*n* = 3). (I) JC-1 staining images showing mitochondrial membrane potential changes. (Scale bar, 25 μm.) (J and K) HMGB1 and ATP levels in 4T1 cell supernatants after different treatments (*n* = 3). Data are presented as mean ± SD (*n* = 3). Statistical significance was analyzed by one-way ANOVA followed by Tukey’s multiple-comparisons test. ns, not significant; *P < 0.05, **P < 0.01, ***P < 0.001, ****P < 0.0001.

To elucidate the tumoricidal mechanism of SCP, cuproptosis-related molecular events and redox imbalance were examined in 4T1 cells. Immunofluorescence staining showed that SCP + NIR treatment markedly reduced FDX1 expression and induced punctate DLAT aggregation, a typical feature associated with copper-induced proteotoxic stress (Fig. 5A to C and Fig. S15) [37]. Consistently, qPCR analysis revealed significant down-regulation of FDX1 and LIAS mRNA in the SCP + NIR group, indicating disruption of the lipoylated protein regulatory machinery (Fig. 5D and E).

SCP + NIR also caused pronounced oxidative stress. 2′,7′-Dichlorodihydrofluorescein diacetate (DCFH-DA) staining and flow cytometry showed a strong intracellular ROS burst after SCP + NIR treatment, exceeding that induced by SCP or NIR alone (Fig. 5F to H and Fig. S16). Meanwhile, JC-1 staining revealed a clear red-to-green fluorescence shift, indicating mitochondrial membrane potential depolarization (Fig. 5I and Fig. S17). Intracellular GSH was also substantially depleted in the SCP + NIR group, further confirming the collapse of antioxidant defenses.

To further verify the involvement of copper-dependent/cuproptosis-associated mechanisms, tetrathiomolybdate (TTM) was used as a copper chelator for inhibitor intervention. TTM alone showed limited cytotoxicity toward 4T1 cells at the selected working concentration, indicating that the inhibitor itself had minimal influence on cell viability. Compared with SCP + NIR treatment alone, TTM intervention reduced intracellular ROS accumulation, as confirmed by DCFH-DA fluorescence imaging and flow cytometry. In addition, TTM partially attenuated SCP + NIR-induced apoptosis/cell death and decreased DLAT punctate aggregation. These inhibitor-based results suggest that copper-dependent/cuproptosis-associated processes contribute to SCP + NIR-induced oxidative damage and tumor cell death (Fig. S18).

Together, FDX1/LIAS down-regulation, DLAT aggregation, ROS accumulation, mitochondrial depolarization, GSH depletion, and TTM-mediated attenuation demonstrate that NIR-activated SCP induces cuproptosis-associated oxidative damage, thereby contributing to its potent antitumor cytotoxicity.

### Induction of ICD hallmarks

To determine whether SCP-induced tumor cell death could elicit immunogenic responses, typical ICD hallmarks were examined in 4T1 cells. As shown in Fig. 5J and K, SCP + NIR treatment markedly enhanced extracellular ATP release and promoted HMGB1 translocation from the nucleus to the extracellular space. Compared with SCP or NIR treatment alone, SCP + NIR induced the strongest DAMP emission, indicating a robust ICD response. These results suggest that NIR-activated SCP not only induces direct tumor cell destruction but also converts dying tumor cells into an immunogenic source, providing a mechanistic basis for subsequent antitumor immune activation in vivo.

### In vivo tumor targeting and precision photothermal performance

**Fig. 6.**
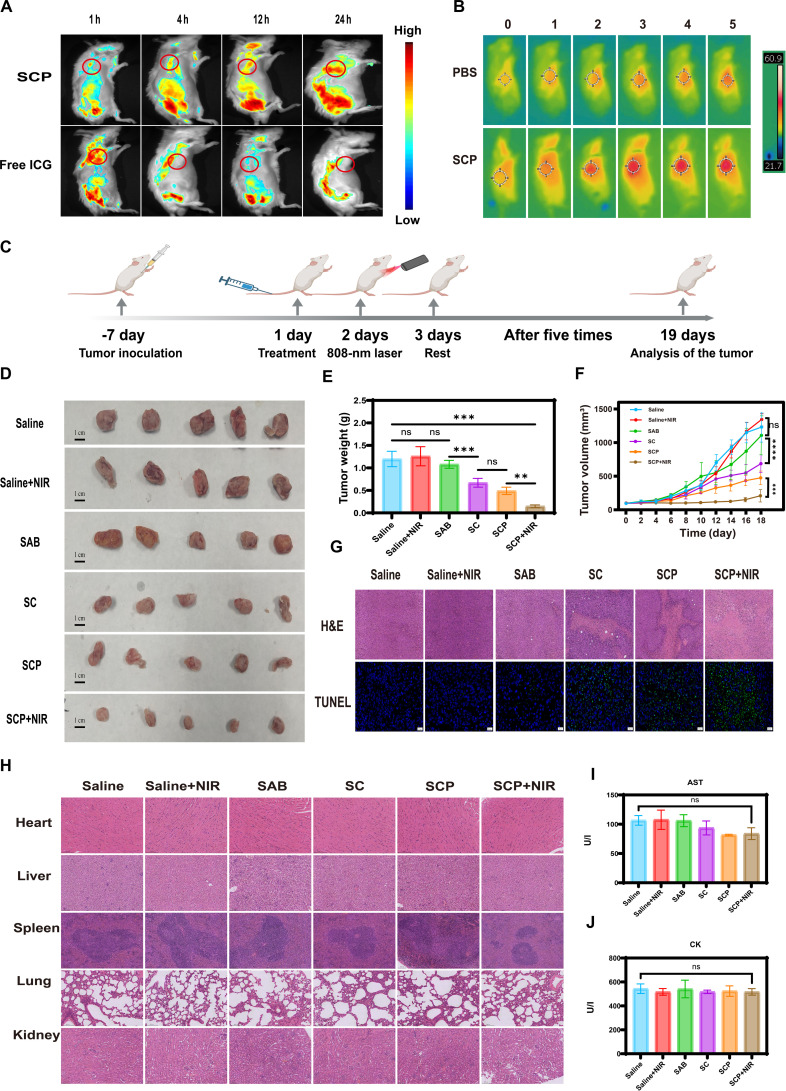
In vivo tumor targeting, photothermal performance, antitumor efficacy, and systemic biosafety of SCP. (A) In vivo fluorescence imaging of tumor-bearing mice at different time points after intravenous injection of free ICG or SCP-ICG. (B) Real-time infrared thermal images of tumor-bearing mice after different treatments under 808-nm NIR irradiation. (C) Schematic illustration of the treatment schedule. (D) Representative tumor photographs after different treatments (*n* = 5). (Scale bar, 1 cm.) (E) Tumor weights after different treatments (*n* = 5). (F) Tumor growth curves from day 1 to day 18 after different treatments (*n* = 5). (G) H&E (scale bar, 100 μm.) and TUNEL (scale bar, 20 μm.) staining images of tumor sections after different treatments. (H) H&E staining images of major organs collected 18 d after different treatments. (Scale bar, 100 μm.) (I and J) Serum biochemical analysis of aspartate aminotransferase (AST) and creatine kinase (CK) levels in different groups (*n* = 3). Data are presented as mean ± SD. Statistical significance in (E), (I), and (J) was analyzed by one-way ANOVA followed by Tukey’s multiple-comparisons test. Statistical significance in (F) was analyzed by 2-way ANOVA followed by Tukey’s multiple-comparisons test. ns, not significant; **P < 0.01, ***P < 0.001, ****P < 0.0001.

The in vivo distribution of SCP was evaluated in 4T1 tumor-bearing mice using ICG-labeled nanoparticles. Fluorescence imaging showed that SCP gradually accumulated at the tumor site after intravenous injection, with the strongest signal observed at 24 h (Fig. 6A and Figs. S19 and S20). This accumulation was likely related to the good colloidal stability of SCP and the enhanced permeability and retention effect. Therefore, 24 h post-injection was selected as the time point for laser irradiation.

The in vivo photothermal effect was then assessed at 24 h after injection. Under 808-nm laser irradiation, the tumor temperature in the SCP group rapidly increased to 52.8 °C within 5 min, whereas the PBS group reached only 43.8 °C under the same conditions (Fig. 6B and Fig. S21). These results indicate that SCP can accumulate in tumors and efficiently convert NIR light into heat, supporting its use for NIR-triggered release and PTT in vivo.

### Potent in vivo antitumor efficacy and systemic biosafety

The antitumor efficacy of the “bridge-and-attack” strategy was evaluated in 4T1 tumor-bearing mice according to the treatment schedule shown in Fig. 6C. Tumor growth was recorded for 18 d (Fig. 6D to F and Fig. S22). PBS and PBS + NIR groups showed rapid tumor progression, suggesting that NIR irradiation alone had minimal therapeutic effect. Free SAB only modestly inhibited tumor growth, with a tumor growth inhibition (TGI) of 18.5%. In comparison, Cu-containing formulations showed stronger antitumor activity. SC achieved a TGI of 51.2%, while SCP further increased the TGI to 67.9%, likely due to the improved colloidal stability and controlled release provided by the PDA shell.

The SCP + NIR group showed the strongest antitumor effect, with a TGI of 88.5% and the lowest final tumor volume and tumor weight. This enhanced efficacy was attributed to the combined effects of NIR-triggered PTT and Cu-mediated cytotoxicity. Consistently, H&E staining showed obvious tumor tissue damage in the SCP + NIR group, including extensive necrotic areas and disrupted cellular structures. TUNEL staining further revealed the highest level of cell apoptosis after SCP + NIR treatment (Fig. 6G).

The biosafety of SCP was also evaluated during treatment. Mice treated with SCP or SCP + NIR showed no obvious body weight loss (Fig. S23), and the major organ indices remained within normal ranges (Fig. S24). H&E staining of major organs showed no apparent histological abnormalities in the heart, liver, spleen, lung, or kidney after SCP or SCP + NIR treatment (Fig. 6H).

Blood biochemical analysis further showed that AST, CK, and CRE levels remained within normal ranges, suggesting no obvious hepatic, cardiac, or renal toxicity (Fig. 6I and J and Fig. S25). These results indicate that SCP + NIR achieves effective tumor inhibition while maintaining good systemic biosafety.

### In vivo orchestration of tumor vascular normalization and hypoxia alleviation

**Fig. 7.**
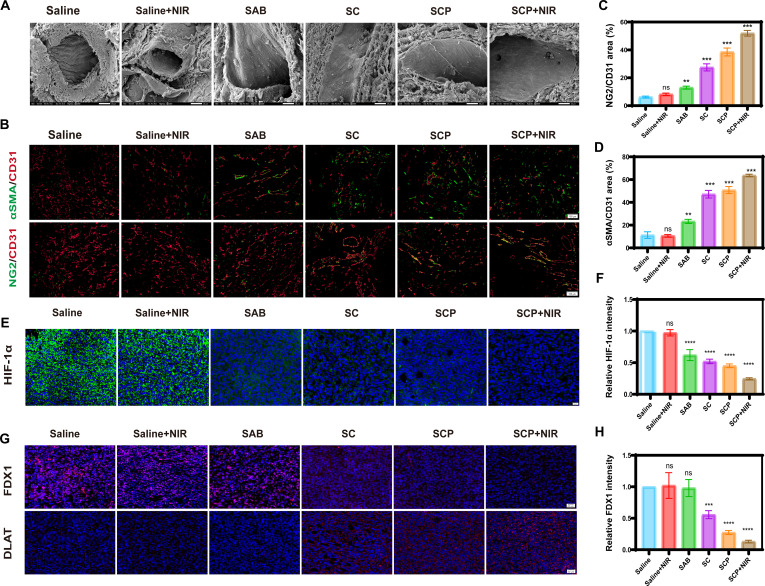
In vivo vascular normalization, hypoxia alleviation, and cuproptosis-associated validation after SCP treatment. (A) Representative scanning electron microscopy (SEM) images of tumor vasculature after different treatments. (Scale bar, 20 μm.) (B) Representative immunofluorescence images of CD31/α-SMA and CD31/NG2 staining in tumor sections after different treatments. (Scale bar, 100 μm.) (C) Quantitative analysis of NG2-positive vessel coverage based on CD31/NG2 immunofluorescence images (*n* = 3). (D) Quantitative analysis of α-SMA-positive vessel coverage based on CD31/α-SMA immunofluorescence images (*n* = 3). (E) Representative HIF-1α immunofluorescence images of tumor sections after different treatments. (Scale bar, 20 μm.) (F) Quantitative analysis of HIF-1α fluorescence intensity (*n* = 3). (G) Representative FDX1 and DLAT immunofluorescence images in tumor sections after different treatments. (Scale bar, 20 μm.) (H) Quantitative analysis of FDX1 fluorescence intensity (*n* = 3). Data are presented as mean ± SD. Statistical significance in (C), (D), (F), and (H) was analyzed by one-way ANOVA followed by Tukey’s multiple-comparisons test. ns, not significant; *P < 0.05, **P < 0.01, ***P < 0.001, ****P < 0.0001

To evaluate the vascular normalization effect, SEM was used to observe the luminal morphology of tumor microvessels (Fig. 7A). Tumors from the Saline and Saline + NIR groups showed disorganized vascular structures, with irregular endothelial arrangement and rough luminal surfaces, indicating dysfunctional tumor vessels [38]. In contrast, SAB-containing treatments improved vascular morphology to different degrees. Compared with free SAB, SCP and SCP + NIR showed more regular endothelial alignment and smoother vessel walls, suggesting improved vascular integrity after treatment.

The maturity of tumor vessels was further assessed by CD31/α-SMA and CD31/NG2 double staining (Fig. 7B). Compared with the control groups, SCP + NIR treatment reduced microvessel density and increased α-SMA^+^ and NG2^+^ pericyte coverage around CD31^+^ vessels (Fig. 7C and D). These results indicate that SCP + NIR promoted tumor vessel maturation and improved vascular stability.

The effect of vascular remodeling on tumor hypoxia was then evaluated by HIF-1α staining. HIF-1α expression was markedly decreased in the SCP + NIR group, suggesting alleviated tumor hypoxia after treatment (Fig. 7E and F). Together, these results demonstrate that SCP enhances the vascular-regulatory effect of SAB and, under NIR irradiation, promotes tumor vascular normalization and hypoxia relief. This improved vascular state may facilitate drug penetration and immune-cell infiltration, supporting the subsequent therapeutic response.

### In vivo mechanistic validation of cuproptosis-driven ICD and systemic immune activation

To verify whether cuproptosis occurred in tumor tissues, FDX1 and DLAT were examined by immunofluorescence staining. Compared with other groups, SCP + NIR treatment markedly reduced FDX1 expression and induced punctate DLAT aggregation (Fig. 7G and H and Fig. S26). These changes are consistent with the disruption of copper-related mitochondrial metabolism and DLAT-associated proteotoxic stress [39,40]. The in vivo results agreed with the cellular experiments, indicating that NIR-activated SCP induced cuproptosis-associated tumor cell damage.

**Fig. 8.**
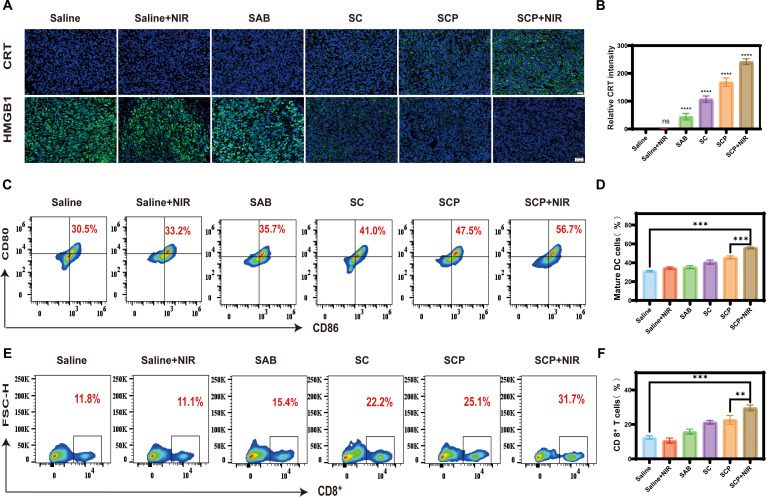
In vivo induction of ICD and antitumor immune activation after SCP treatment. (A) Representative immunofluorescence images of CRT and HMGB1 staining in tumor sections after different treatments. (Scale bar, 20 μm.) (B) Quantitative analysis of CRT fluorescence intensity in tumor sections (*n* = 3). (C) Representative flow cytometry plots of mature DCs, defined as CD80^+^CD86^+^ cells gated on CD11c^+^ DCs, in tumor-draining lymph nodes after different treatments. (D) Quantitative analysis of mature DCs (*n* = 3). (E) Representative flow cytometry plots of CD8^+^ T cells in tumor tissues after different treatments. (F) Quantitative analysis of CD8^+^ T cells (*n* = 3). Data are presented as mean ± SD. Statistical significance in (B), (D), and (F) was analyzed by one-way ANOVA followed by Tukey’s multiple-comparisons test. ns, not significant; **P < 0.01, ***P < 0.001, ****P < 0.0001.

ICD-related markers were then evaluated in tumor tissues. SCP + NIR treatment increased CRT exposure and reduced nuclear HMGB1 staining (Fig. 8A and B and Fig. S27), suggesting HMGB1 translocation/release and ICD induction. These results indicate that SCP + NIR not only caused tumor cell death but also promoted immunogenic signals within the TME.

Flow cytometry was further used to assess immune activation. SCP + NIR treatment increased the proportion of mature DCs (CD80^+^CD86^+^) to 56.7% in tumors and 25.1% in spleens, which was higher than that in the other groups (*P* < 0.001, Fig. 8C to F). Consistently, CD8^+^ T cells increased to 31.7% in tumors and 30.8% in spleens after SCP + NIR treatment (Figs. S28 and S29). These results suggest that local ICD was accompanied by DC maturation and CD8^+^ T cell activation.

Together, these findings show that SCP + NIR promotes cuproptosis-associated ICD and enhances antitumor immune activation in vivo. This immune response, together with vascular normalization and PTT, contributes to the improved therapeutic effect against TNBC.

## Discussion and Conclusion

TNBC remains a major clinical challenge because of its high metastatic potential, limited therapeutic targets, and poor response to conventional treatments [41]. These difficulties are closely associated with its immunosuppressive “cold” TME [42]. In particular, abnormal tumor vasculature limits nanomedicine extravasation and penetration, while hypoxia further weakens immune-cell function and antitumor responses [43]. Therefore, improving vascular function before applying cytotoxic therapy may provide a practical strategy to enhance TNBC treatment.

In this study, we developed a PDA-coated, NIR-responsive copper–polyphenol nanoplatform, SCP, to realize a NIR-triggered “bridge-and-attack” strategy. Upon NIR irradiation, SCP promoted the co-release of SAB and copper ions, thereby activating 2 coupled therapeutic effects. SAB served as the “bridge” component by promoting vascular normalization, as shown by improved endothelial morphology, increased pericyte coverage, and reduced tumor hypoxia. Meanwhile, released copper ions, together with PDA-mediated photothermal activation, acted as the “attack” component by inducing Cu^+^/Cu^2+^-mediated redox stress, DLAT-associated cuproptosis, and ICD. As a result, SCP + NIR achieved an 88.5% tumor growth inhibition rate in vivo and increased CD8^+^ T cell infiltration, indicating effective remodeling of the TNBC immune microenvironment.

The “bridge” and “attack” effects should be interpreted according to their different biological time scales. NIR-triggered photothermal activation and copper release occur rapidly after irradiation and mainly account for the immediate tumor cell killing through local heating, ROS generation, and cuproptosis-associated damage. In contrast, SAB-mediated vascular normalization requires progressive remodeling of the tumor vasculature, including improved endothelial integrity, increased pericyte coverage, enhanced perfusion, and hypoxia alleviation. Thus, SAB release is expected to progressively establish and maintain a vascular normalization window during repeated treatment cycles, thereby improving subsequent nanoparticle delivery, oxygenation, and immune-cell infiltration, rather than instantaneously enhancing the same-cycle Cu^2+^/photothermal effect.

A key finding of this study is that SCP improved tumor vascular structure rather than simply suppressing angiogenesis. Conventional anti-angiogenic therapies, such as vascular endothelial growth factor (VEGF) blockade, can reduce vessel density but may also aggravate hypoxia when vascular pruning is excessive or prolonged, thereby limiting therapeutic efficacy and promoting resistance [44]. In contrast, SAB released from SCP promoted a vascular normalization-like effect in tumors. SEM observation showed more regular vascular morphology after SCP treatment, while CD31/NG2 and CD31/α-SMA staining confirmed increased pericyte and smooth muscle cell coverage around tumor vessels. These changes indicate improved vessel maturation and stability. Consistently, HIF-1α expression was reduced, suggesting alleviated tumor hypoxia. This normalized vascular state may enhance intratumoral delivery of copper ions and photothermal agents while also supporting immune-cell infiltration. Thus, the SAB-mediated “bridge” effect supported copper- and photothermal-mediated tumor cell killing and contributed to the overall antitumor response.

Upon NIR irradiation, SCP initiated the “attack” component through copper-mediated cell damage and PDA-mediated PTT. Compared with therapies mainly relying on apoptosis, cuproptosis provides an alternative way to induce tumor cell death through copper-dependent proteotoxic stress. In this study, NIR irradiation enhanced copper release from SCP and promoted PDA-mediated heating. The released copper was associated with reduced FDX1 expression and DLAT aggregation, indicating disturbance of the lipoylated tricarboxylic acid (TCA)-cycle protein machinery and activation of cuproptosis-related damage.

In addition, SCP further amplified intracellular oxidative stress. The Cu^+^/Cu^2+^ redox cycle promoted Fenton-like ROS generation, while GSH depletion weakened the antioxidant defense of tumor cells. Together with photothermal heating, these effects led to mitochondrial dysfunction and increased tumor cell death. This combined mechanism, including PTT, chemodynamic activity, and cuproptosis-associated damage, helps explain the strong tumor inhibition observed in vivo, with a TGI of 88.5% in the SCP + NIR group.

The coupled effects of SAB-mediated vascular normalization, copper-associated tumor cell damage, and PDA-mediated PTT also contributed to immune microenvironment remodeling [45]. TNBC is often poorly infiltrated by effector lymphocytes, which limits the response to immunotherapy. In this study, SCP + NIR treatment induced typical ICD signals, including CRT exposure and the release or translocation of HMGB1 and ATP. These changes can promote antigen presentation and help initiate antitumor immune responses. The vascular normalization effect may further support this immune activation. By improving vessel maturity and reducing hypoxia, SCP created a more favorable microenvironment for immune-cell infiltration. Flow cytometry showed increased maturation of DCs in both tumors and spleens, together with enhanced CD8^+^ T cell infiltration after SCP + NIR treatment. These results suggest that SCP + NIR not only induces local tumor cell death but also promotes antitumor immune activation. Therefore, the integration of vascular normalization, PTT, cuproptosis-associated damage, and ICD provides a coordinated strategy for remodeling the TNBC microenvironment.

Taken together, to further clarify the superiority of SCP resulting from these coordinated mechanisms, we compared our system with representative copper-based cuproptosis/photothermal nanoplatforms and metal–phenolic network systems reported in recent literature (Table S1). Previous strategies predominantly targeted tumor cell-intrinsic death pathways, failing to address the vascular barriers that restrict nanomedicine extravasation and T cell infiltration [46–49]. Conversely, SCP tackles this by integrating a vascular “bridge” with a dual-mode “attack” within one bioinspired platform. Quantitatively, SCP demonstrated superior photothermal conversion (59.5%) and highly controlled, TME-responsive copper release (a 3.53-fold increase under pH 6.5 + NIR versus pH 7.4). By successfully translating these efficient physicochemical properties in vivo, SCP expanded pericyte coverage to 51.6% and alleviated hypoxia. This normalized vascular route markedly enhanced intratumoral nanoparticle delivery and immune-cell trafficking, resulting in robust DC maturation, substantial CD8^+^ T cell recruitment, and an 88.5% tumor inhibition rate. Thus, the distinction of SCP lies in its capability for coordinated vascular normalization and microenvironment remodeling.

Beyond antitumor efficacy, SCP also showed good systemic biosafety. During treatment, mice in the SCP and SCP + NIR groups maintained stable body weight, and major organ indices remained within normal ranges. H&E staining revealed no obvious pathological changes in the heart, liver, spleen, lung, or kidney after SCP-based treatments. Blood biochemical analysis further showed that AST, CK, and CRE levels remained within normal ranges, suggesting no apparent hepatic, cardiac, or renal toxicity. This safety profile may be related to the PDA shell, which improves colloidal stability and reduces premature cargo leakage during circulation, as well as the preferential tumor accumulation observed in the biodistribution study. These results support the potential of SCP for systemic administration.

In conclusion, we developed a bioinspired PDA-coated copper–polyphenol nanoplatform, SCP, for NIR-triggered “bridge-and-attack” therapy against TNBC. Upon NIR irradiation, SCP promoted the co-release of SAB and copper ions, enabling coupled vascular remodeling and tumor cell killing. SAB-mediated vascular normalization increased pericyte coverage and alleviated tumor hypoxia, thereby supporting subsequent nanoparticle delivery and immune-cell infiltration. Meanwhile, released copper ions and PDA-mediated photothermal activation induced Cu^+^/Cu^2+^-associated oxidative stress, GSH depletion, mitochondrial dysfunction, DLAT-associated cuproptosis, and ICD. As a result, SCP + NIR enhanced DC maturation, increased CD8^+^ T cell infiltration, and achieved an 88.5% tumor inhibition rate in 4T1 tumor-bearing mice with favorable systemic biosafety. Further studies on long-term clearance, pharmacokinetics, and validation in more clinically relevant models are still needed. Overall, this work provides a practical biomaterial-mediated strategy for integrating vascular normalization with copper-based photothermal immunotherapy for TNBC treatment.

## Ethical Approval

All in vivo experiments were conducted in accordance with the guidelines for the care and use of laboratory animals established by the National Institutes of Health (NIH), and were performed in a pathogen-free environment at the animal facility of Tianjin University of Traditional Chinese Medicine. The animal protocols were approved by the Institutional Animal Ethics Committee of Tianjin University of Traditional Chinese Medicine (document number: TCM-LAEC2025218S2067).

## Data Availability

The data supporting the findings of this study are available from the corresponding authors upon reasonable request.
