# Supplementary material for: Near-Infrared-Triggered “Bridge-and-Attack” Strategy via a Bioinspired Copper–Polyphenol Nanoarchitectonics Platform for Vascular Normalization-Enhanced Cuproptosis-Immunotherapy of Triple-Negative Breast Cancer
Source: Biomater Res. 2026 Aug 3;30:0398. doi: 10.34133/bmr.0398 (PMC13429914; doi:10.34133/bmr.0398)
Supplement: Supplementary 1 — Figs. S1 to S29 Table S1 [file bmr.0398.f1.docx]

**Supporting Information**

**Materials**

**A**

**
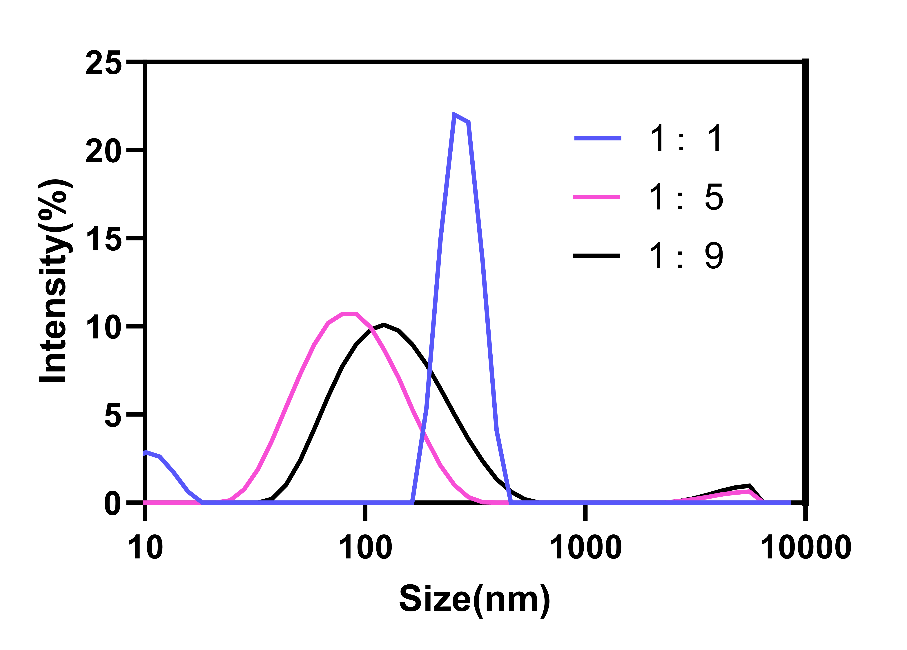
**

**B**

| SAB:Cu²⁺ | Size (nm) | PDI | Cu EE (%) | SAB EE (%) |
| --- | --- | --- | --- | --- |
| 1:1 | 1979 | 0.939 | N.D. | N.D. |
| 1:5 | 99.0 | 0.171 | 73 | 80 |
| 1:9 | 122.6 | 0.257 | 86 | 78 |

**Supplementary Figure 1 Optimization of the SAB:Cu²⁺ feeding ratio for SC nanoassembly formation.** (A) DLS size distribution curves of SC prepared at different SAB:Cu²⁺ feeding ratios. (B) Summary of particle size, PDI, and encapsulation efficiencies of Cu and SAB at different feeding ratios. N.D., not determined due to severe aggregation and poor colloidal stability. The ratios indicate the feeding mass ratios of SAB to Cu²⁺.

**A**

**
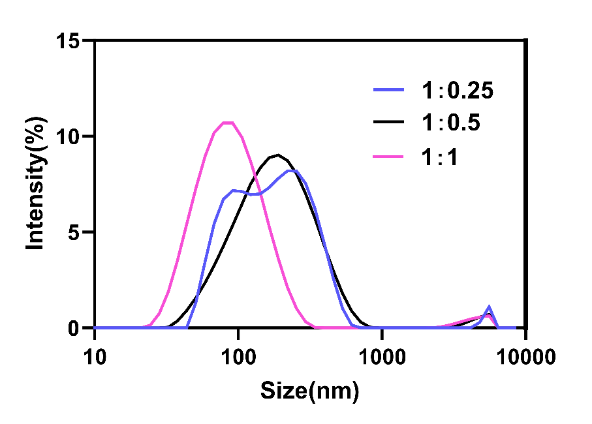
**

**B**

**
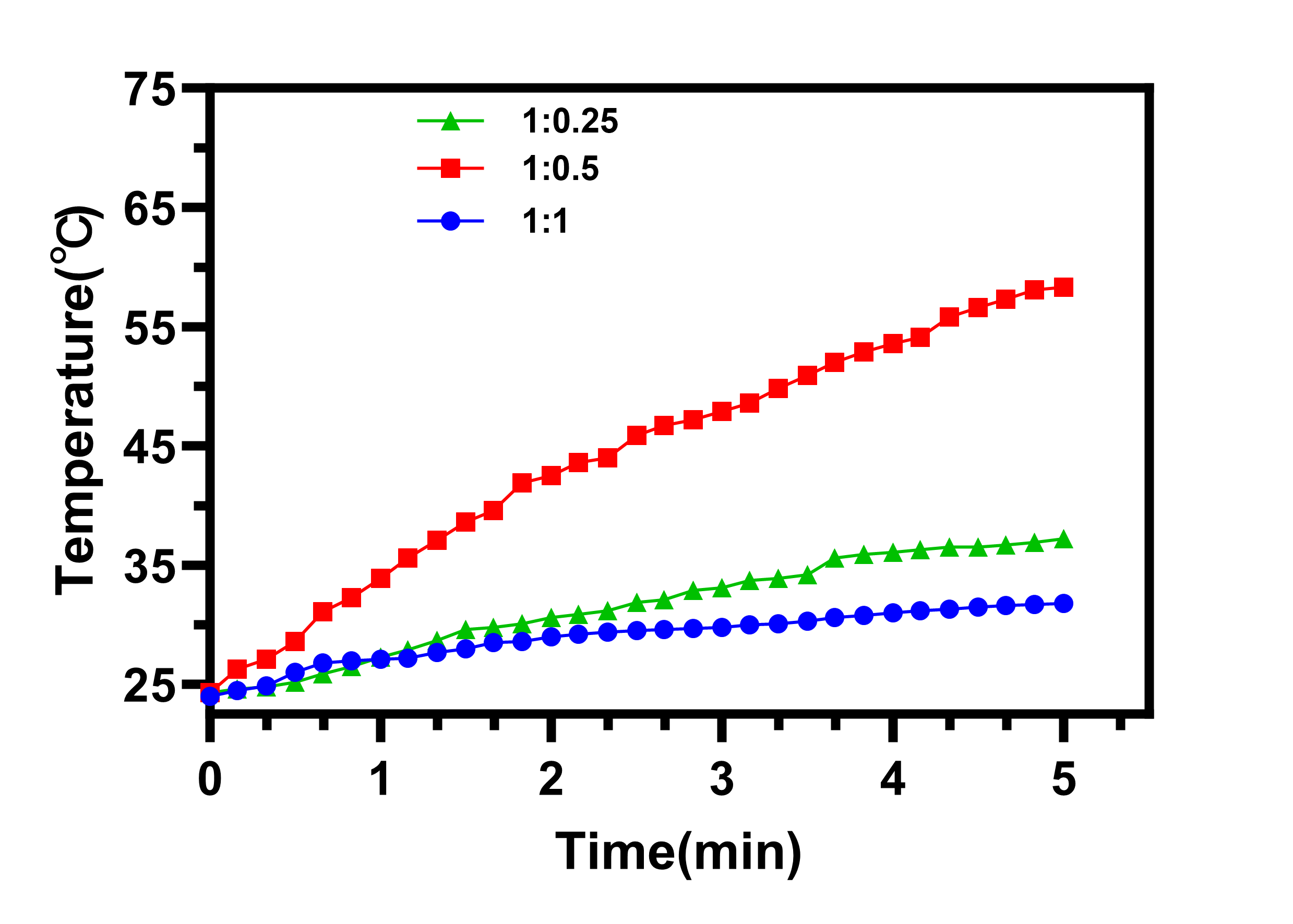
**

**C**

**
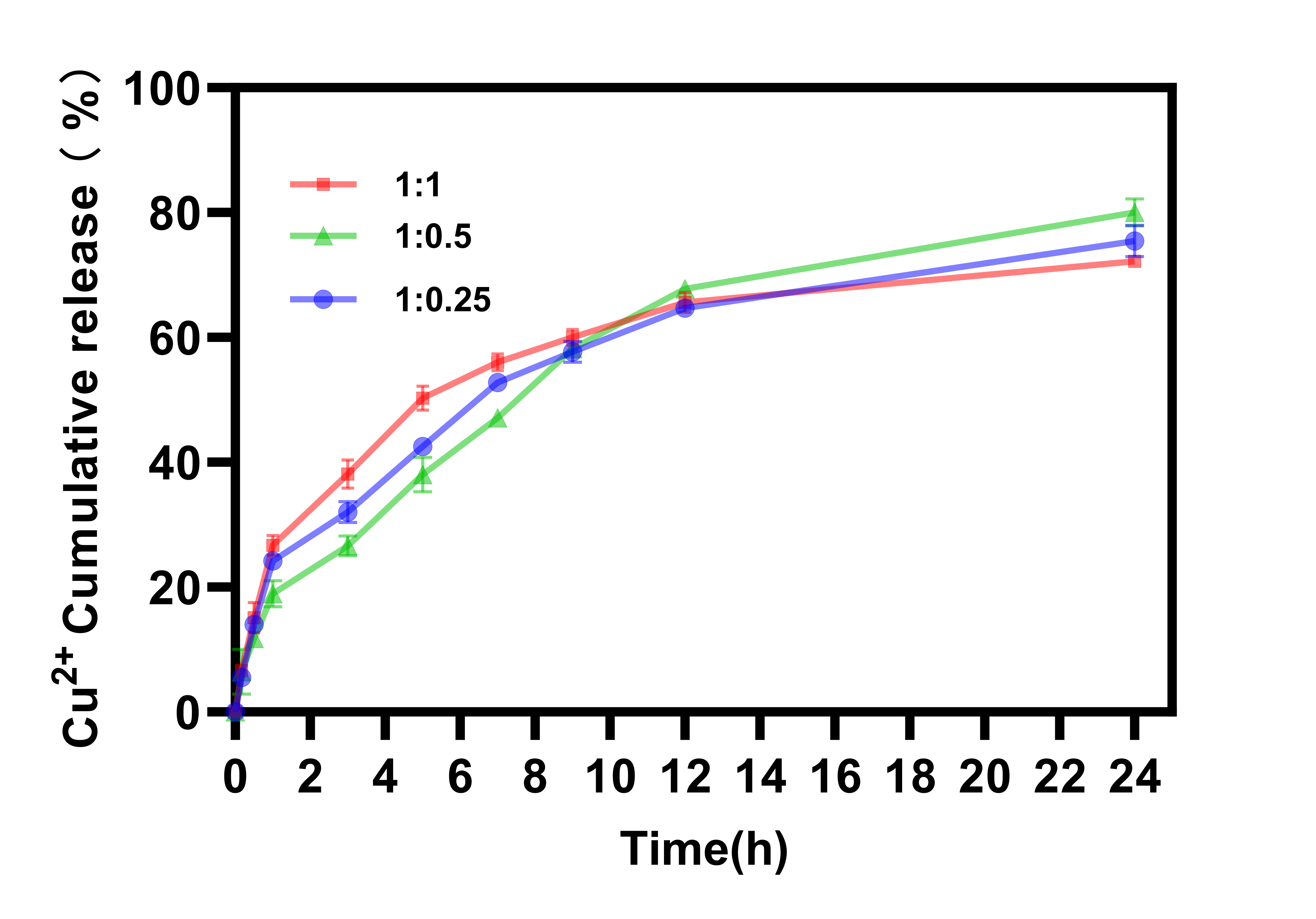
**

**Supplementary Figure 2. Optimization of the dopamine feeding ratio for PDA coating during SCP preparation.** (A) DLS size distribution curves of SCP prepared with different dopamine feeding ratios. (B) Photothermal heating curves of SCP prepared with different dopamine feeding ratios under 808 nm laser irradiation. (C) Cu²⁺ cumulative release profiles of SCP prepared with different dopamine feeding ratios under the indicated release condition. The ratios indicate the feeding mass ratios of SAB to dopamine.


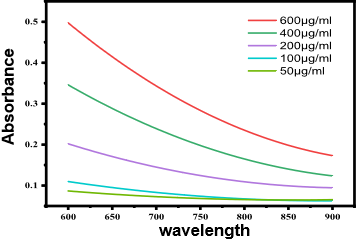


**Supplementary Figure 3. UV–vis–NIR absorption spectra of SCP at different concentrations.** SCP dispersions with increasing concentrations exhibited concentration-dependent absorbance in the NIR region, supporting their potential for photothermal conversion under 808 nm laser irradiation.


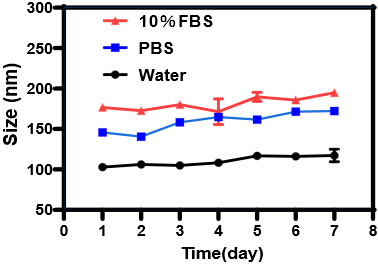


**Supplementary Figure 4. Colloidal stability of SCP under different storage media.** Hydrodynamic size changes of SCP were monitored in deionized water, PBS, and 10% FBS over 7 days to evaluate colloidal stability under aqueous, physiological buffer, and serum-containing conditions. Data are presented as mean ± SD (n = 3).


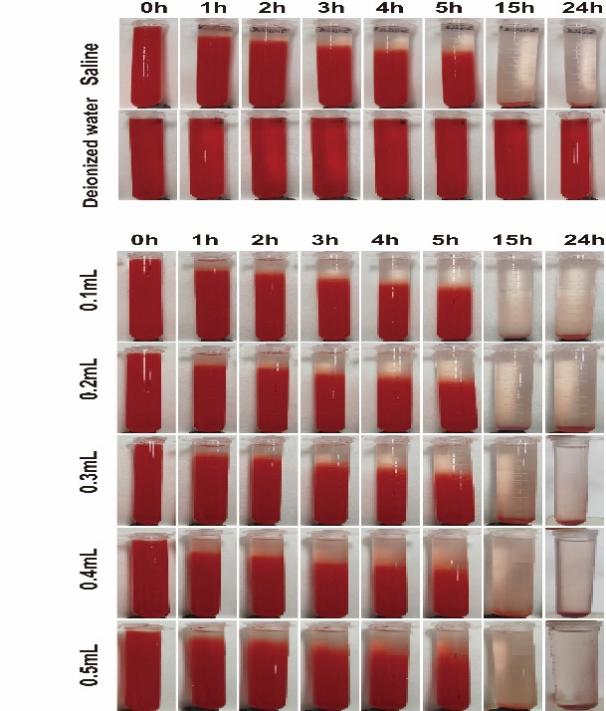


**Supplementary Figure 5. In vitro hemolysis assay of SCP.** Photographs of erythrocyte suspensions after incubation with SCP at the indicated concentrations and time points. Distilled water and saline were used as positive and negative controls, respectively, to assess the hemocompatibility of SCP.


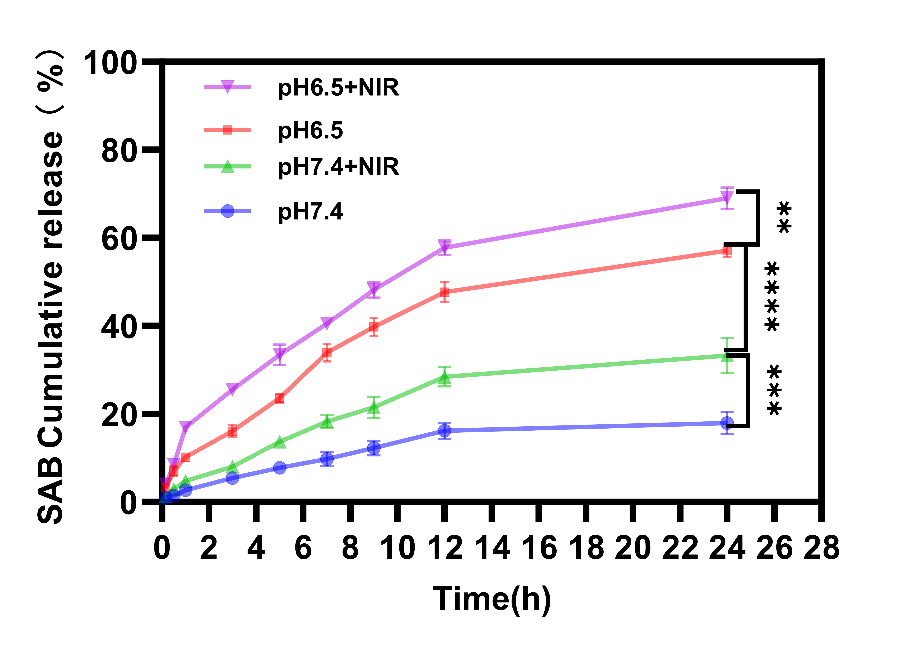


**Supplementary Figure 6. In vitro SAB release profile from SCP under different pH and NIR irradiation conditions.** Cumulative SAB release profiles from SCP at pH 7.4 and pH 6.5 with or without 808 nm NIR irradiation (n = 3). Acidic pH and NIR irradiation promoted SAB release, indicating the pH/NIR dual-responsive release behavior of SCP. Data are presented as mean ± SD (n = 3). Statistical significance was analyzed by two-way ANOVA followed by Tukey’s multiple-comparisons test. Statistical comparisons were performed at 24 h; *p < 0.05, **p < 0.01, ***p < 0.001, ****p < 0.0001.


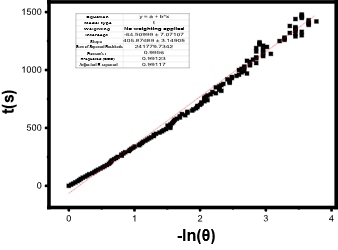


**Supplementary Figure 7. Linear fitting curve for calculating the photothermal conversion efficiency of SCP.** The linear relationship between cooling time and −ln(θ) was obtained from the cooling period after 808 nm laser irradiation. The fitted slope was used to calculate the heat transfer time constant and photothermal conversion efficiency.


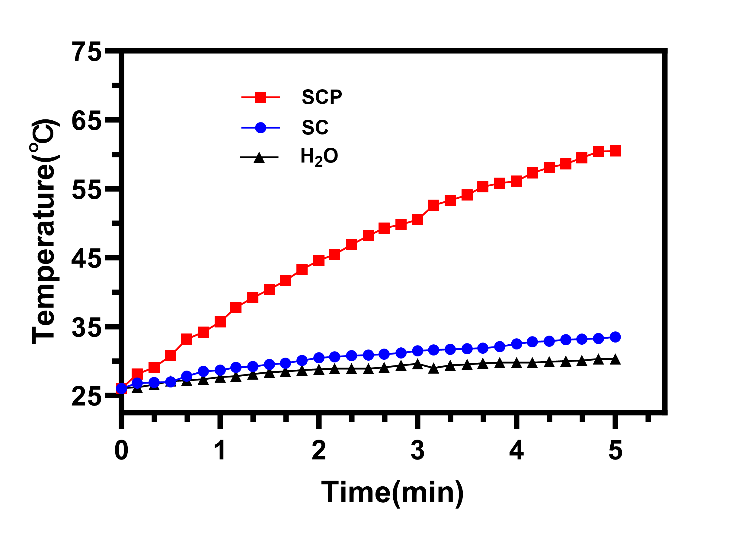


**Supplementary Figure 8. Photothermal heating comparison of PDA-free SC and PDA-coated SCP.** Photothermal heating curves of H₂O, SC, and SCP under 808 nm laser irradiation at 2 W/cm². SC and SCP were tested at the same concentration of 400 μg/mL.


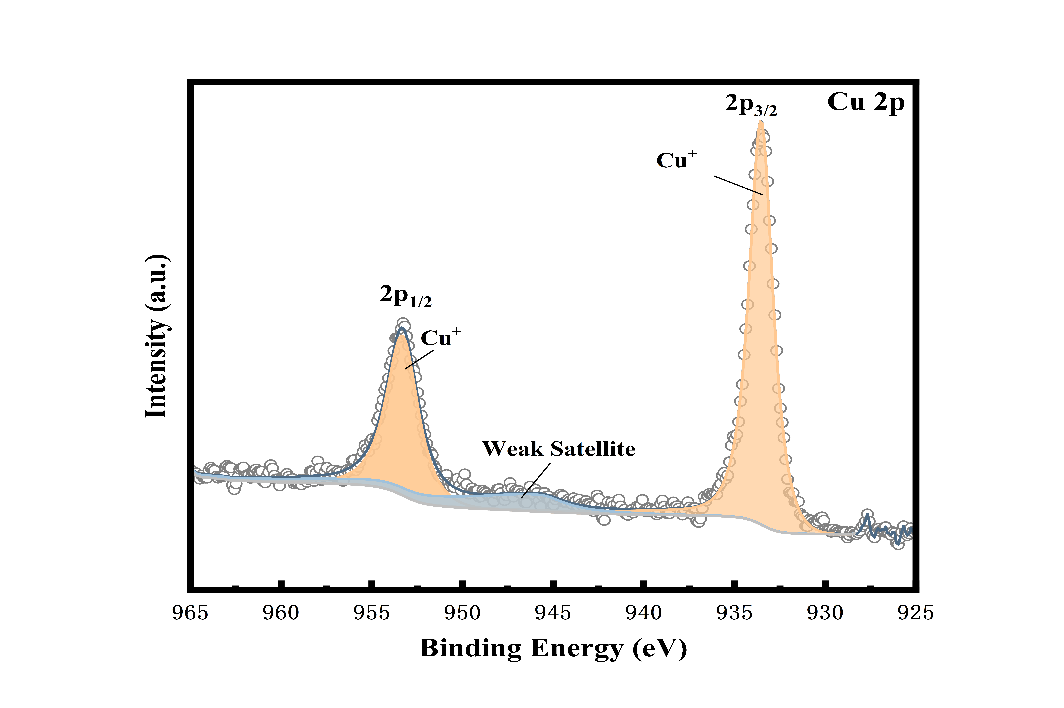


**Supplementary Figure 9. High-resolution XPS spectrum of Cu 2p in SCP.** Cu 2p₃/₂ and Cu 2p₁/₂ peaks were analyzed to determine the valence state of copper in SCP and to verify the presence of Cu species in the coordination nanoassembly.


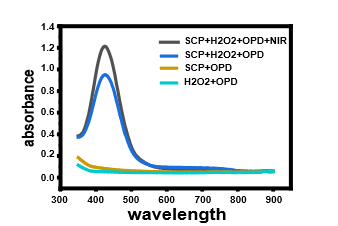


**Supplementary Figure 10. Evaluation of Fenton-like catalytic activity of SCP using OPD as a chromogenic probe.** UV–vis absorption spectra of OPD after different treatments, including H₂O₂ + OPD, SCP + OPD, SCP + H₂O₂ + OPD, and SCP + H₂O₂ + OPD + NIR. The increased absorbance at approximately 417 nm indicates enhanced oxidation of OPD and ·OH generation, which was further promoted by NIR irradiation.


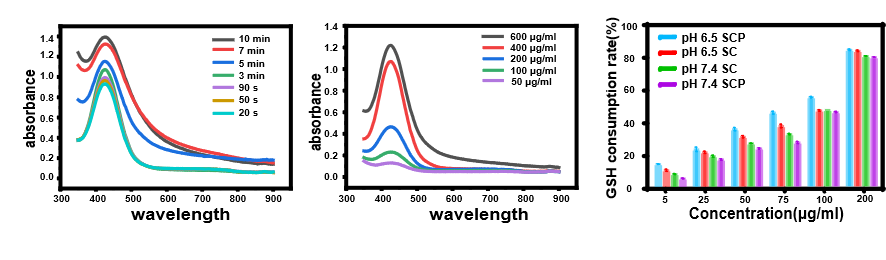


**Supplementary Figure 11. ROS generation and GSH depletion ability of SCP.** UV–vis absorption spectra were recorded to evaluate ROS generation under different irradiation times and SCP concentrations. GSH consumption rates of SC and SCP were further measured under pH 6.5 and pH 7.4 conditions to assess pH-dependent GSH depletion. Data are presented as mean ± SD (n = 3).

**Supplementary Figure 12. Quantitative analysis of Rhodamine B fluorescence intensity in 4T1 cells after incubation with Rhodamine B-labeled SCP for 2 h or 4 h with or without NIR irradiation.** Data are presented as mean ± SD (n = 3). Statistical significance was analyzed by one-way ANOVA followed by Dunnett’s multiple-comparisons test, with each group compared with the SCP 2 h group. *p < 0.05, ****p < 0.0001.


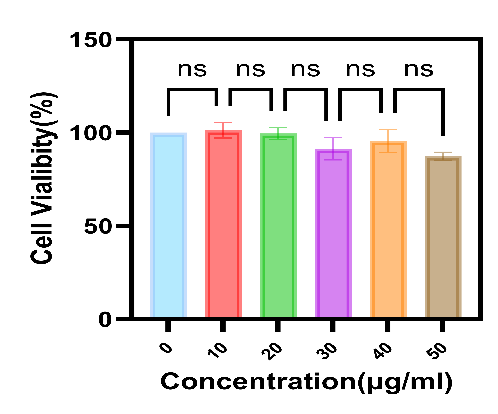


**Supplementary Figure 13. Cytocompatibility of SCP in 3T3 cells.** Cell viability of 3T3 cells after incubation with SCP at different concentrations for 24 h. Data are presented as mean ± SD (n = 3). Statistical significance was analyzed by one-way ANOVA followed by Tukey’s multiple-comparisons test, with each treatment group compared with the untreated control group. ns, not significant.

**Supplementary Figure 14.** Quantitative analysis of the PI/Calcein-AM fluorescence ratio in 4T1 cells after different treatments based on live/dead staining images (n = 3). Data are presented as mean ± SD.

**Supplementary Figure 15. Quantitative analysis of FDX1 fluorescence intensity in 4T1 cells after different treatments.** Data are presented as mean ± SD (n = 3). Statistical significance was analyzed by one-way ANOVA followed by Dunnett’s multiple-comparisons test, with each treatment group compared with the Control group. ns, not significant; ****p < 0.0001.

**Supplementary Figure 16. Quantitative analysis of intracellular ROS levels based on DCF fluorescence intensity in 4T1 cells after different treatments.** Data are presented as mean ± SD (n = 3). Statistical significance was analyzed by one-way ANOVA followed by Dunnett’s multiple-comparisons test, with each treatment group compared with the Control group. ns, not significant; ****p < 0.0001.

**Supplementary Figure 17. Quantitative analysis of mitochondrial membrane potential based on the JC-1 aggregate/monomer fluorescence ratio in 4T1 cells after different treatments.** A decreased JC-1 aggregate/monomer ratio indicates mitochondrial membrane potential depolarization. Data are presented as mean ± SD (n = 3). Statistical significance was analyzed by one-way ANOVA followed by Dunnett’s multiple-comparisons test, with each treatment group compared with the Control group. ns, not significant; ****p < 0.0001.


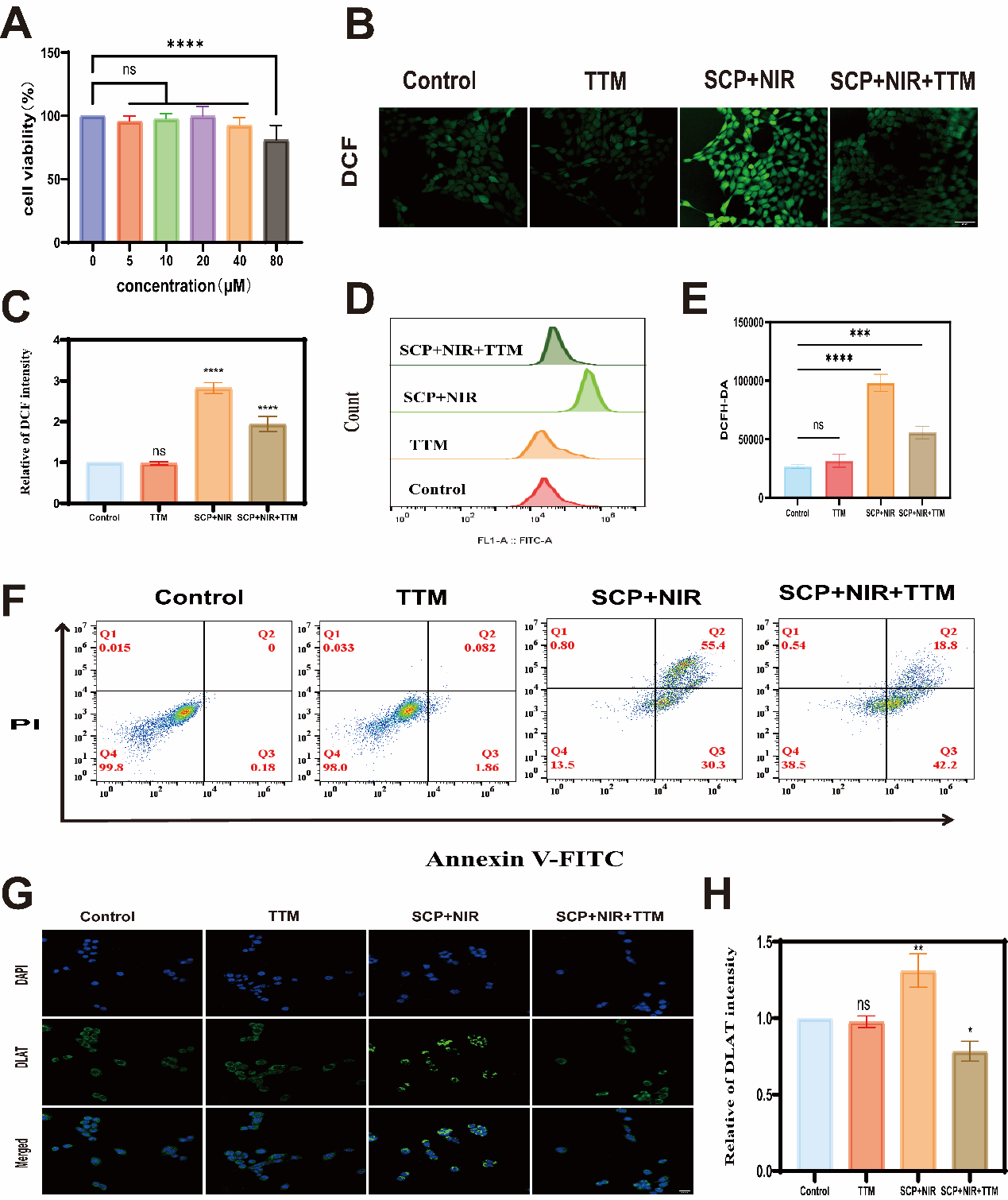


**Supplementary Figure 18. TTM intervention attenuates SCP+NIR-induced ROS generation, apoptosis, and DLAT aggregation in 4T1 cells.** (A) Cell viability of 4T1 cells after treatment with different concentrations of tetrathiomolybdate (TTM) for 24 h. (B) Representative DCFH-DA fluorescence images showing intracellular ROS generation in 4T1 cells after different treatments. (Scale bar = 50 μm.) (C) Quantitative analysis of DCF fluorescence intensity based on fluorescence images (n = 3). (D) Representative flow cytometry histograms of DCF fluorescence after different treatments. (E) Quantitative analysis of intracellular ROS levels based on DCF mean fluorescence intensity (MFI) (n = 3). (F) Representative Annexin V-FITC/PI flow cytometry plots of 4T1 cells after different treatments. (G) Representative DLAT immunofluorescence images in 4T1 cells after different treatments. (Scale bar = 50 μm.) (H) Quantitative analysis of DLAT fluorescence intensity based on confocal images (n = 3). Data are presented as mean ± SD. Statistical significance was analyzed by one-way ANOVA followed by Tukey’s multiple-comparisons test. For the TTM cytocompatibility assay in panel A, statistical significance was analyzed by one-way ANOVA followed by Dunnett’s multiple-comparisons test, with each TTM-treated group compared with the untreated control group. ns, not significant; *p < 0.05, **p < 0.01, ***p < 0.001, ****p < 0.0001.

**
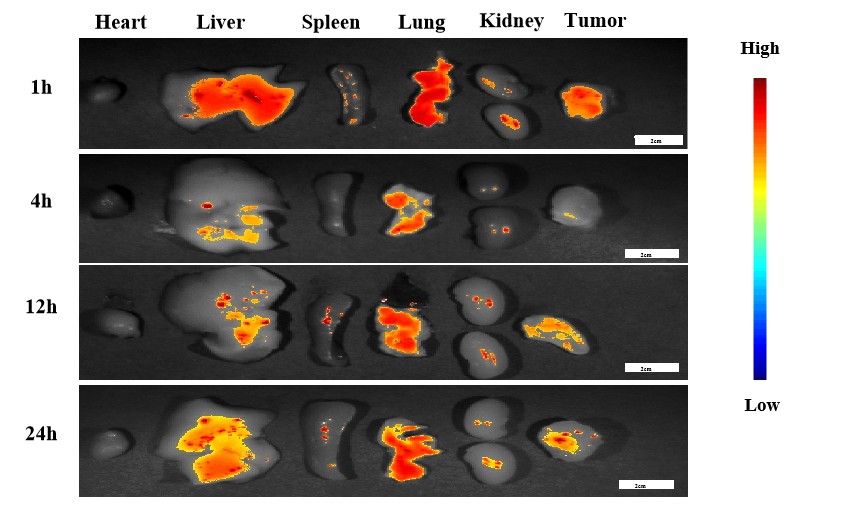
**

**Supplementary Figure 19. Ex vivo fluorescence imaging of major organs and tumors after intravenous injection of SCP-ICG.** Heart, liver, spleen, lung, kidney, and tumor tissues were collected at the indicated time points after injection to evaluate the biodistribution and tumor accumulation of SCP. (scale bar=2cm)


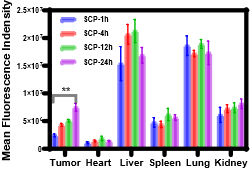


**Supplementary Figure 20. Quantitative analysis of ex vivo fluorescence intensity in major organs and tumors after SCP-ICG injection.** Mean fluorescence intensity was quantified from heart, liver, spleen, lung, kidney, and tumor tissues at 1, 4, 12, and 24 h post-injection. Data are presented as mean ± SD (n = 3). **P < 0.01.


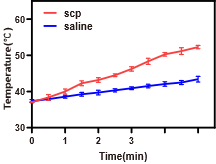


**Supplementary Figure 21. In vivo tumor temperature changes after intravenous injection of saline or SCP.** Tumor-bearing mice were irradiated with an 808 nm NIR laser after administration, and tumor surface temperature was monitored over time using an infrared thermal imaging system. Data are presented as mean ± SD (n = 3).


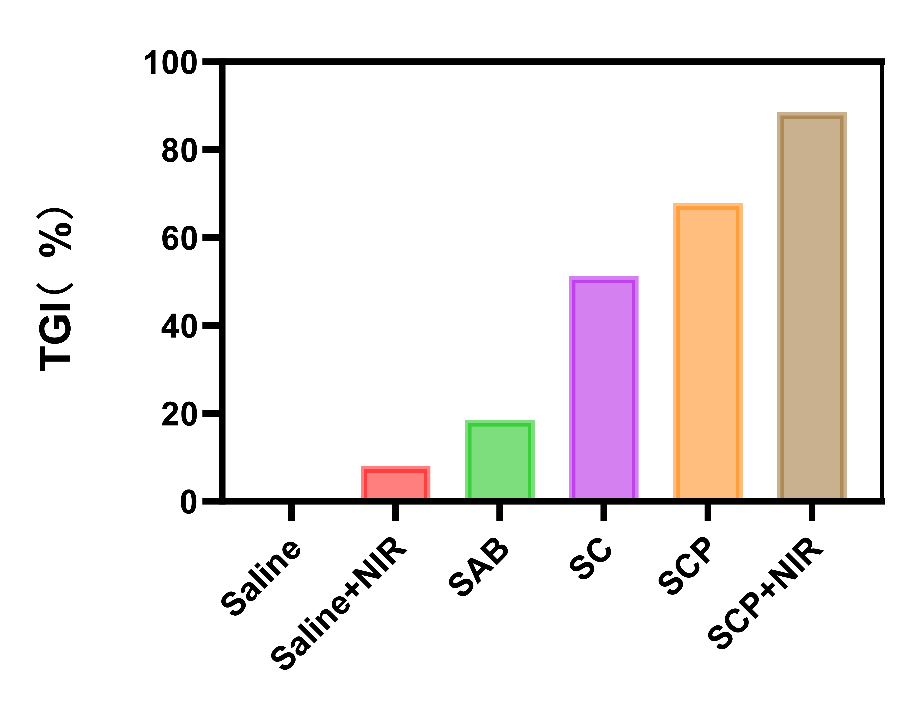


**Supplementary Figure 22. Tumor growth inhibition rate (TGI) after different treatments.** TGI was calculated based on the final tumor weight in each treatment group, including saline, saline + NIR, SAB, SC, SCP, and SCP + NIR.


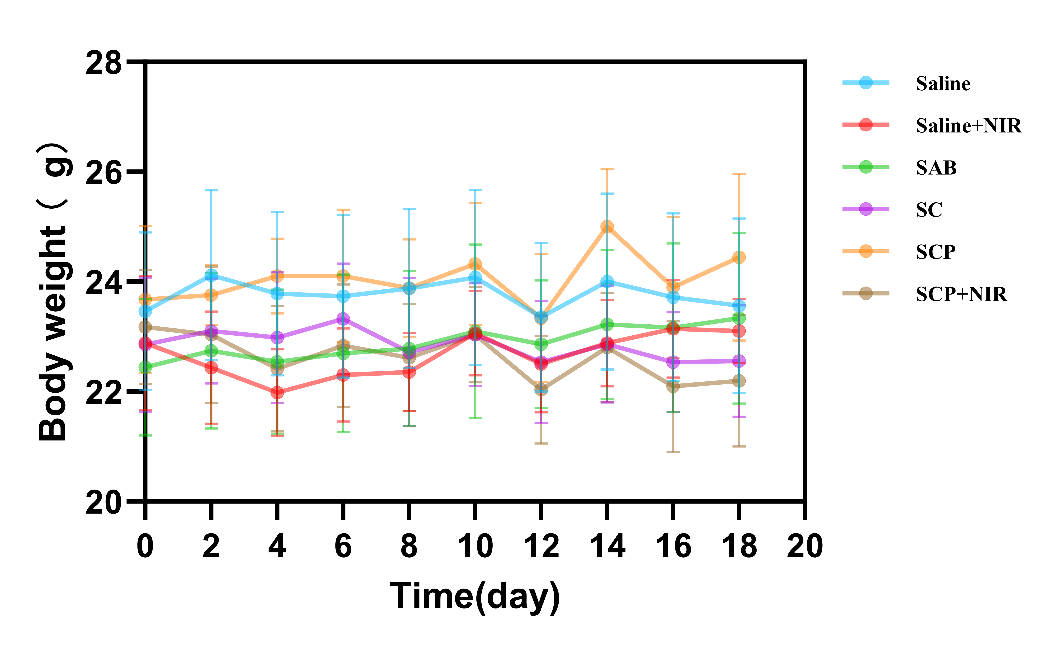


**Supplementary Figure 23. Body weight changes of mice during treatment.** Body weights of tumor-bearing mice in different treatment groups were monitored throughout the treatment period to evaluate systemic tolerability. Data are presented as mean ± SD (n = 5).

**
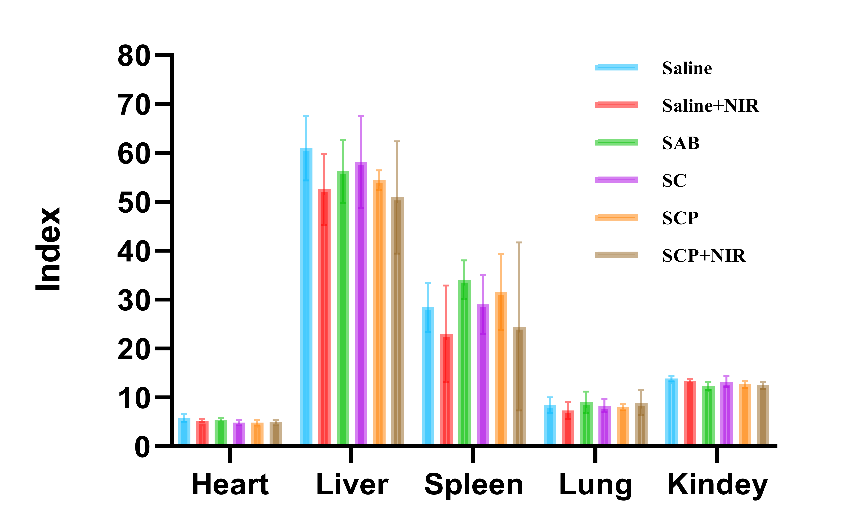
**

**Supplementary Figure 24. Major organ indices of mice after different treatments.** Organ indices of heart, liver, spleen, lung, and kidney were calculated after treatment to evaluate potential systemic toxicity and organ-level safety. Data are presented as mean ± SD (n = 5).


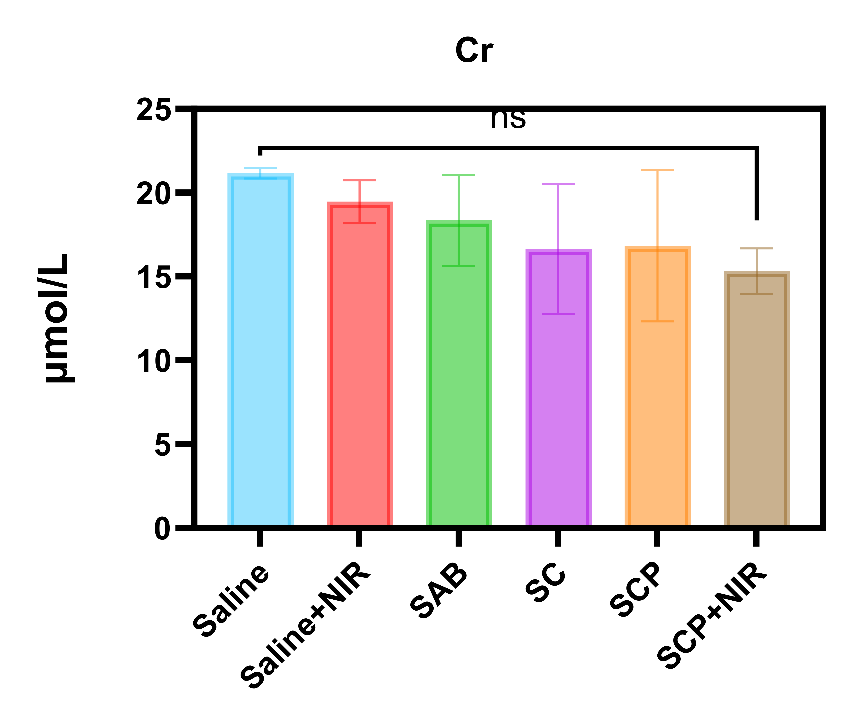


**Supplementary Figure 25. Serum creatinine levels after different treatments.** Serum creatinine (CR) was measured to evaluate renal function in mice receiving saline, saline + NIR, SAB, SC, SCP, or SCP + NIR treatment. Data are presented as mean ± SD (n = 3). ns, not significant.

**Supplementary Figure 26. Quantitative analysis of DLAT fluorescence intensity in tumor sections after different treatments (n = 3).** Data are presented as mean ± SD. Statistical significance was analyzed by one-way ANOVA followed by Dunnett’s multiple-comparisons test, with each treatment group compared with the Saline group. ns, not significant; ****p < 0.0001.

**Supplementary Figure 27. Quantitative analysis of HMGB1 fluorescence intensity in tumor sections after different treatments.** Data are presented as mean ± SD (n = 3). Statistical significance was analyzed by one-way ANOVA followed by Dunnett’s multiple-comparisons test, with each treatment group compared with the Saline group. ns, not significant; **p < 0.01, ****p < 0.0001.


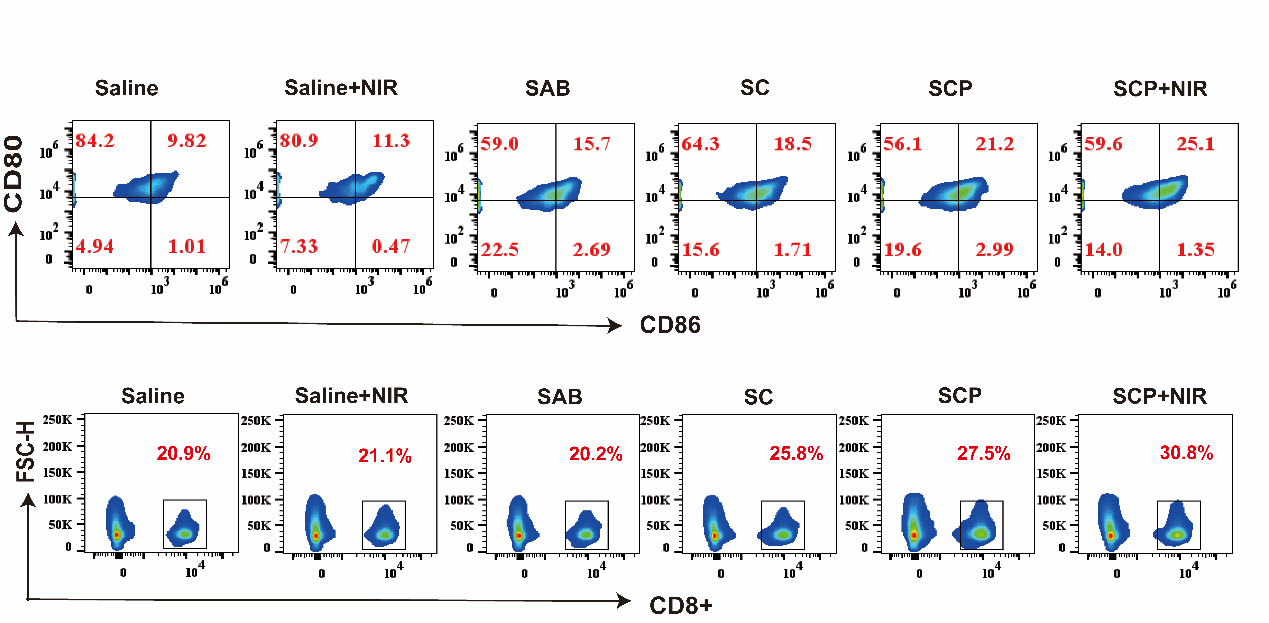


**Supplementary Figure 28. Representative flow cytometric plots of splenic mature DCs and CD8⁺ T cells.** Mature dendritic cells were identified as CD80⁺CD86⁺ cells, and CD8⁺ T cells were analyzed in spleen samples from mice receiving different treatments, including saline, saline + NIR, SAB, SC, SCP, and SCP + NIR.


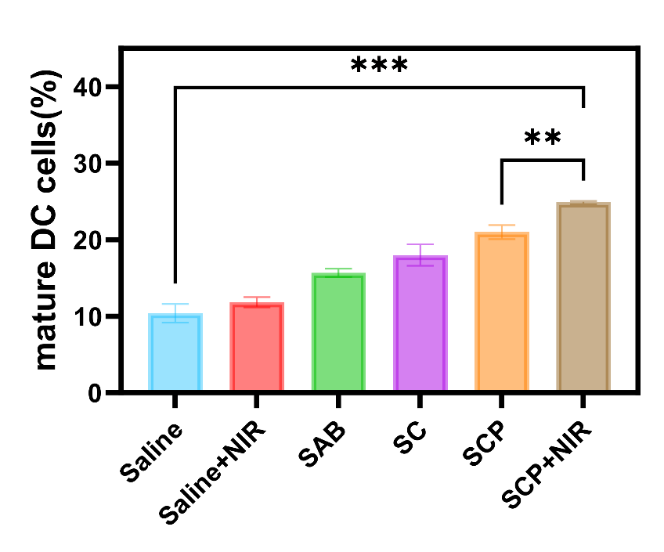

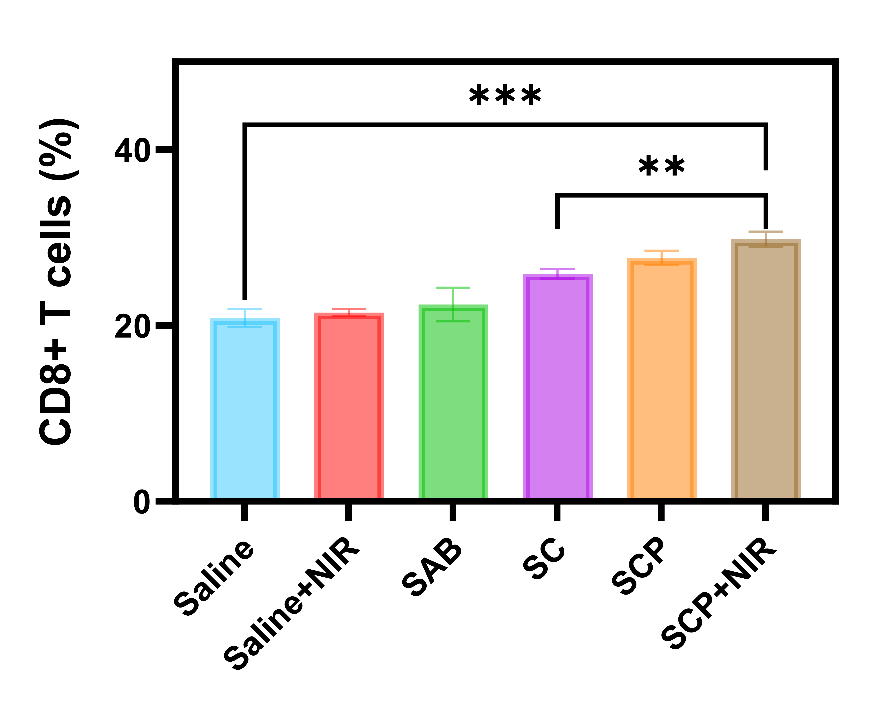


**Supplementary Figure 29. Quantitative analysis of splenic mature DCs and CD8⁺ T cells after different treatments.** The percentages of mature DCs and CD8⁺ T cells were quantified from flow cytometry data to evaluate systemic immune activation. Data are presented as mean ± SD (n = 3). Statistical significance was determined by one-way ANOVA with multiple-comparison analysis; **P < 0.01, ***P < 0.001.

**Supplementary Table S1. Comparison of SCP with representative reported copper-based or vascular-remodeling nanoplatforms.**

| **System** | **Main design** | **Major therapeutic mechanism** | **Representative quantitative features** | **Difference from SCP** |
| --- | --- | --- | --- | --- |
| Cu-GA metal–phenolic network NPs [46] | Cu ions coordinated with gallic acid | GSH-responsive Cu release, apoptosis/cuproptosis and chemodynamic therapy | GSH-triggered degradation and Cu⁺/GA release were reported | Mainly focuses on redox/cell-death regulation; vascular normalization and NIR-triggered release were not emphasized |
| CuSACO single-atom nanozyme [47] | Bioorthogonal Cu single-atom nanozyme | Nanozyme catalytic therapy, PTT, cuproptosis and immunotherapy | Efficient inhibition of orthotopic breast tumors, gliomas and lung metastasis was reported | Strong catalytic/PTT platform, but vascular “bridge” remodeling was not a core design |
| CAPSH[48] | Cu₉S₈/AIPH/siATP7A/HA nanoplatform | NIR-II photothermal therapy, alkyl-radical generation, ATP7A silencing and cuproptosis | Tumor DCs and CD8⁺ T cells increased to 15.5% and 20.8%, respectively | Potently regulates copper homeostasis, but mainly targets tumor-cell-intrinsic copper efflux rather than vascular normalization |
| T-T@Cu  [49] | Carrier-free metal–phenolic copper nanoplatform | Mitochondrial copper delivery, Cys/GSH depletion, cuproptosis/ferroptosis and mild PTT | Photothermal conversion efficiency ≈48.5%; 4T1 IC₅₀ ≈12.87 μg/mL; tumor inhibition ≈100% | Very strong tumor-cell killing, but vascular normalization and immune-cell trafficking improvement were not the central mechanism |
| SCP, this work | SAB-Cu coordination core coated with PDA | SAB-mediated vascular normalization + NIR-triggered Cu release/PTT + cuproptosis/ICD | Cu²⁺ release: 20.7% at pH 7.4 vs 73.08% at pH 6.5+NIR; η=59.5%; pericyte coverage=51.6%;mature DCs=56.7%; CD8⁺ T cells=31.7%; TGI=88.5% | Integrates vascular “bridge”, controlled copper release, high photothermal activation and immune remodeling in one platform |

Note. Data for reported systems were extracted from the corresponding literature. Because different studies used different tumor models, dosing regimens, laser parameters, and evaluation time points, this table is intended to compare representative mechanistic and quantitative features rather than to provide a direct head-to-head efficacy ranking.
